# Families Moving Forward Connect mHealth Intervention for Caregivers of Children With Fetal Alcohol Spectrum Disorders: Randomized Controlled Trial

**DOI:** 10.2196/73647

**Published:** 2026-03-20

**Authors:** Christie L M Petrenko, Madeline N Rockhold, Julianne M Myers, Cristiano Tapparello, Carson Kautz-Turnbull, Emily Speybroeck, Zhi Li, Lynn L Cole, Heather Carmichael Olson

**Affiliations:** 1 Mt. Hope Family Center, University of Rochester, 187 Edinburgh St., Rochester, NY, 14608, United States, 1 585-275-2991 ext 241, 1 585-454-2972; 2 Division of Developmental and Behavioral Pediatrics, University of Rochester, Rochester, NY, United States; 3 Department of Electrical and Computer Engineering, University of Rochester, Rochester, NY, United States; 4 Department of Psychiatry and Behavioral Sciences, Center on Child Health, Behavior and Development, School of Medicine, University of Washington, Seattle, WA, United States; 5 Seattle Children's Research Institute, Seattle, WA, United States

**Keywords:** fetal alcohol spectrum disorders, prenatal alcohol exposure, mobile health, intervention, treatment, parenting, families moving forward, neurodevelopmental disabilities, mobile phone

## Abstract

**Background:**

Fetal alcohol spectrum disorders (FASD) affect 1.1% to 5% of the general population. Yet, most children with FASD and their families cannot access evidence-based interventions. Mobile health (mHealth) interventions have the potential to increase access to care on a broad scale. While numerous self-directed parenting apps exist, none have been tested for FASD. The FMF (Families Moving Forward) Connect app is a self-directed intervention derived from an empirically supported intervention for caregivers raising children with FASD. FMF Connect is the first self-directed parenting app for FASD, and also one of the first parenting apps to be systematically developed and tested.

**Objective:**

This study aimed to test the efficacy of FMF Connect for caregivers raising children with FASD on targeted primary (child behavior, caregiver attributions, parenting efficacy and satisfaction, FASD knowledge, and family needs met) and secondary (child adaptive behavior, caregiver self-care, and app satisfaction) outcomes.

**Methods:**

This study involved a 3-arm randomized controlled trial with equal allocation to groups (1) FMF Connect+coaching, (2) FMF Connect, or (3) waitlist control. Participants from the United States were recruited online through an open access website. Recruitment materials were distributed by the Collaborative Initiative on FASD, FASD listserves, and social media. In total, 129 caregivers of children (aged 3‐12 y) with FASD or prenatal alcohol exposure (PAE) were enrolled. Online surveys were administered at baseline, 6 weeks, and 12 weeks. Data were analyzed with linear mixed modeling, linear regressions, and structural equation modeling using SPSS (version 29.0; IBM) and Mplus 8 (Muthén & Muthén).

**Results:**

A total of 43 participants were randomized to each group. Caregivers were predominantly White adoptive mothers. Of the total, 64% (n=83) of participants were retained through the 12-week follow-up. Groups did not differ in terms of demographic characteristics, baseline levels of functioning, or attrition. Usage patterns were similar across groups, suggesting coaching did not increase engagement. Given a few differences, app intervention groups were combined for analyses. Relative to the waitlist group, caregivers in the FMF Connect group evidenced greater improvements in FASD knowledge, child behavior attributions, family needs met, and self-care after 12 weeks (*P*=.01-.048). After controlling for multiple comparisons, differences in FASD knowledge, self-care, and family needs met approached significance (*P*=.06-.07). Groups did not differ in parenting satisfaction, child behavior problems, or adaptive functioning. More app usage is related to greater changes in parenting efficacy. Caregiver behavior attributions at 6 weeks did not mediate intervention effects.

**Conclusions:**

This study demonstrated initial efficacy of the FMF Connect app for targeted caregiver outcomes, with small to medium effect sizes. As an mHealth app, the FMF Connect intervention has potential for scalability and accessibility. This could lead to a substantial public health impact, particularly for families who face challenges accessing evidence-based resources or encounter other barriers to care.

## Introduction

Fetal alcohol spectrum disorders (FASD) are highly prevalent developmental disabilities. Studies estimate that FASD affects 1.1%‐5% of the population [1]. Yet, most people with FASD remain undiagnosed and have inadequate access to services [2]. Diagnosis of FASD focuses on evidence of prenatal alcohol exposure (PAE) and neurobehavioral impairment. Additional symptoms, such as microcephaly, seizures, subtle characteristic facial features, and growth delays, are present in approximately 10% of individuals with FASD [1,3-5]. Individuals with FASD often require lifelong, multidisciplinary, community-based services in order to receive appropriate diagnosis, treatment planning, and daily-living supports [6-9]. Caregivers of children with FASD experience high rates of stress, feelings of isolation, and relationship strain between child and caregiver [7,10,11]. Despite higher needs, individuals with FASD and their families report substantial barriers to services due to limited availability of knowledgeable providers, societal stigma, lack of policy-based supports, and insufficient availability of appropriate, evidence-based services [9,12]. These systematic barriers emphasize the need for evidence-based interventions that are both accessible and tailored to the needs of families managing FASD, presenting an opportunity to apply innovative methods and new implementation strategies.

One method to surmount these barriers is mobile delivery of services or app-based interventions. There has been an increase in technology-augmented parenting programs that have been shown to support intervention engagement, caregiver skill building, and positive child behavior outcomes [13-15]. While promising, these programs require additional live therapeutic services and many are web-based. This trend does not reflect current accessibility and preference patterns, which indicate higher preference for mobile devices compared with desktop services [16]. Most adults have access to smartphones and demonstrate consistent understanding and use of phone-based apps [17]. Mobile health (mHealth) apps provide self-paced access to material, services, and supports that can be more easily scaled to larger populations than in-person or telehealth programs [18]. A recent meta-analysis documented that health behavior apps can result in significant improvement in behavior change across a variety of settings [19]. This suggests that app-based interventions are a promising way to solve problems of intervention accessibility.

Despite the promise of app-based interventions, there have been very few mHealth apps with evidence-based review that follows recommended mental health app guidelines [20]. A meta-review of parenting-based technological interventions by David and colleagues [13] indicated that of the 53 usable parenting-focused apps available in app stores, few offered personalized content or focused on caregiver functioning, and none have yet been evaluated in a randomized controlled trial (RCT).

As mHealth apps offer a promising solution to many barriers faced by families and providers when trying to access FASD-informed care, the FMF (Families Moving Forward) Connect app was developed to provide accessible, evidence-based support for caregivers of children with FASD. The FMF Connect app is the first known stand-alone, self-directed parenting app to be empirically evaluated for efficacy for caregiver and child outcomes [21-23]. The app was derived from the FMF Program, a 14- to 17-session program for caregivers of children aged 3‐12 years with FASD and challenging behavior problems delivered by a trained specialist [24-26]. This program was developed and tested by Olson and colleagues [24-26] at Seattle Children’s Research Institute and the University of Washington, with funding from the Centers for Disease Control and Prevention. The FMF Program was designed to address the needs of diverse family structures, socioeconomic status, and racial and ethnic identities, in clinic, home-based, or, recently, telehealth settings. Derived from developmental and family systems theories, the FMF Program integrates positive behavior support, motivational interviewing, and cognitive behavioral techniques. The FMF Program helps caregivers to understand their child’s strengths and challenges and alter cognitions and attitudes toward child behavior from that of willful disobedience to a neurodevelopmental “reframed” approach. Using this neurodevelopmental viewpoint, families are encouraged to focus on accommodations to support their children’s needs and are also helped to understand behavior management. Research has shown that the FMF Program offers benefits for both caregivers and children [24,27,28] (personal communication by HCO, March 5, 2016, and March 2, 2017). Among other findings, caregivers experience improvements in their knowledge of FASD and advocacy skills, parenting efficacy, and some specialized parenting practices. Additionally, they report better self-care and a greater sense of their family’s needs being met. For these developmentally complex children, the program leads to positive changes in behavior immediately post treatment. A growing number of innovative programs are being derived from the FMF Program to meet the diverse needs of families, affected children and youth of different ages, and various settings [21-23,29,30]. This includes the FMF Connect caregiver app, which is the focus of this study.

**Figure 1.**
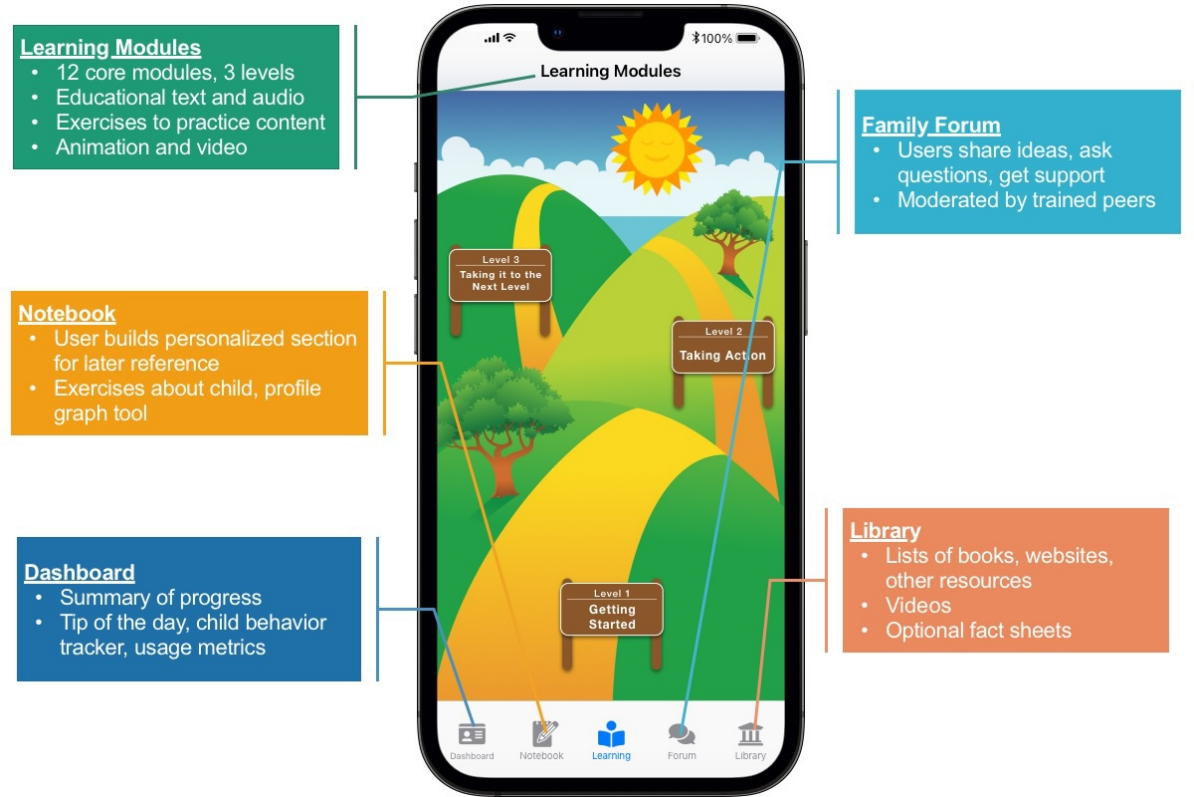
The 5 main components of the Families Moving Forward (FMF) Connect app: Learning Modules, Library, Notebook, Family Forum, and Dashboard.

The FMF Connect app incorporates core content, principles, and methods of the standard FMF Program while also adding new features and content to support caregivers using the app in a self-directed, digital format. FMF Connect consists of 5 primary components, as illustrated in Figure 1, Learning Modules, Library, Notebook, Family Forum, and Dashboard. A coaching component was also trialed in this study to see if text-based coaching could increase engagement with the app and improve family outcomes.

The systematic user-centered design approach to develop and test the FMF Connect app has been relatively rare in the field of mHealth apps. This approach, detailed in the study by Petrenko et al [22], emphasizes iterative feedback at each stage of development from community members who represent target users for the app. This process has included focus groups with caregivers on the initial design and functionalities of the proposed app [23], interviews with caregivers and providers following beta testing of initial app prototypes [22], and surveys and interviews with caregivers during a larger-scale feasibility trial [21]. This study represents the next critical phase of our systematic evaluation, which is to test the efficacy of the app in an RCT. The primary objective of this study was to determine if the FMF Connect app was related to improved targeted caregiver and child outcomes over a 12-week period. This study was conducted in the United States and involved a 3-arm parallel RCT with equal allocation to 3 study groups who received (1) FMF Connect app plus text-based coaching to support app engagement, (2) FMF Connect app without coaching, and (3) waitlist control (WLC) group who received the app after study conclusion. Relative to the waitlist control group, we hypothesized that assignment to the FMF Connect groups would be associated with increases in caregiver FASD knowledge, improvements in parenting efficacy and satisfaction, reductions in caregiver attributions of willful child behavior, increases in family needs met, and improved child behavior. We hypothesized that the addition of text-based coaching would result in greater app usage and, in turn, greater change across outcomes. A 6-week time point was also included to test whether improvements in caregiver attributions of child behavior would mediate intervention effects on child behavior and caregiver self-efficacy and satisfaction. We also assessed caregiver perceptions of app quality, consistent with previous feasibility trials [21].

## Methods

### Ethical Considerations

This study was registered at ClinicalTrials.gov on August 24, 2021, before study initiation. The full study protocol and study consent document are publicly available within the ClinicalTrials.gov study record. All study procedures and materials were reviewed and approved by the University Institutional Review Board (STUDY00006555). Deidentified data were coded by participant number and identifying information was securely stored separately. There were no costs to participants for participating in the study. They received payments for completing surveys (US $40 at baseline, US $20 at 6 weeks, and US $40 at 12 weeks).

### Trial Design

This study involved a 3-arm parallel RCT with equal allocation to the following conditions: (1) FMF Connect+Coaching, (2) FMF Connect without coaching, and (3) waitlist control. Quantitative survey data were collected at 3 time points—baseline, 6 weeks, and 12 weeks. The 6-week time point involved an abbreviated battery designed to allow for tests of statistical mediation hypotheses. App usage was recorded through Amazon Web Services (AWS) during the study period. After 12 weeks, study participants in the waitlist control group received the FMF Connect app. No changes were made to the trial design after initiation.

### Participants

The participant population included caregivers of children (aged 3‐12 y) with FASD or PAE who live in the United States. To be eligible, caregivers needed to be a biological parent or other primary caregiver (eg, foster or adoptive parent, relative, or legal guardian) who was at least 18 years old, living in the United States, and had a smartphone or iPad (Apple Inc) with iOS operating system. Caregivers needed to have a child between the ages of 3 and 12 years with a diagnosis of FASD or confirmed PAE. As part of study registration, caregivers completed a survey about the child’s PAE and uploaded diagnostic records, if available. If there was more than 1 child in the family with FASD or PAE in this age range, the caregiver was instructed during screening to choose 1 child as the target of study surveys. The child needed to have lived with the caregiver for at least 4 months and was expected to remain in the home for at least 1 year. Exclusion criteria included the caregiver not being fluent in English (the FMF Connect app and measures were only available in English). Caregivers were also excluded if there was another caregiver of the same child or living in the home who was already enrolled in the study (couples are excluded to prevent dependence in the data), the caregiver participated in a previous trial of the FMF Connect app as part of earlier development phases of this project [21-23], or the family had previously received or was currently receiving the specialist-led standard FMF Program.

Recruitment, informed consent, and data collection procedures were conducted online. Study recruitment strategy and materials were developed through consultation with the University’s Clinical and Translational Science Institute, with considerations to engage a diverse participant pool. These considerations included materials representing diverse caregivers and families across race, ethnicity, age, and family structures and strategies to leverage partnerships and social media (ie, Facebook [Meta] and Twitter [subsequently rebranded as X]) outlets across the United States. Participants were recruited through a variety of mechanisms by our study team and collaborators within the Collaborative Initiative on FASD (CIFASD; [31]) and community partners. These included disseminating recruitment materials on social media, through national and regional FASD listserves (eg, FASD United’s Weekly Round-up), at relevant conferences, and to participants or patients with whom the study team or collaborators had routine access and/or had agreed to contact about new research opportunities. We aimed to enroll about 150 participants in this study. Power analyses were conducted before recruitment using GPower3. This analysis determined that with a sample size of 120 and assuming α=.05, there will be a power of 0.97 to detect medium effects (f^2^=0.15) and power >0.99 to detect large effects (f^2^=0.35). We anticipated about 20% attribution based on the previous feasibility trial [21].

Study recruitment materials included links to the study website [32], where potential participants could access study information and link to the study’s consenting and screening module. All surveys were administered via REDCap (Research Electronic Data Capture), hosted by the University of Rochester [33,34], and REDCap’s eConsent package was used to document participants’ informed consent. Participants had the option to review study consent information by video or text and were required to answer several questions to ensure comprehension. Participants were also offered the opportunity to ask questions via direct contact with the study coordinator before proceeding to other parts of the survey.

After completing informed consent, participants were directed in REDCap to answer screening questions to assess for study eligibility, including information about PAE, FASD diagnostic records, and demographic information. The study team determined eligibility using a study-created eligibility verification form in REDCap to carefully review the criteria and records provided. No evidence of bots or fraudulent participants was detected. A globally unique identifier [35] was then assigned to each eligible caregiver participant and child to facilitate data sharing within CIFASD or future studies. Eligible participants then received an email inviting them to complete baseline study surveys.

### Outcomes

Table 1 lists all study surveys administered per timepoint. Primary study outcomes included child behavior problems (the Eyberg Child Behavior Inventory [ECBI]), attributions of child behavior (the Reasons for Children’s Behavior Scale [RCB]), parenting self-efficacy and satisfaction (the Parenting Sense of Competence Scale [PSOC]), family needs met (the Family Needs Met [FNM] Questionnaire), and caregiver FASD knowledge (the FASD Knowledge and Advocacy [K&A] Questionnaire). Secondary outcomes included child adaptive functioning (the Everyday Life Scale [ELS]) and caregiver use of self-care. We also examined participant ratings of app quality (the User Version of the Mobile Health Application Rating Scale [uMARS]) and patterns of app usage (from AWS). Additional measures of family background and services were included to characterize the sample. There were no changes in measurement after study initiation.

**Table 1.** Study assessment battery.

Measure	Baseline	6 weeks	12 weeks
Eyberg Child Behavior Inventory (ECBI)	✓		✓
Reasons for Children’s Behavior Scale (RCB)	✓	✓	✓
Parenting Sense of Competence Scale (PSOC)	✓	✓	✓
Family Needs Questionnaire (FNM)	✓		✓
Knowledge & Advocacy Questionnaire (K&A)	✓		✓
Everyday Life Scale (ELS)	✓	✓	✓
Self-Care and Support Assessment	✓		✓
Family Background & Services Survey	✓		✓
User Version of the Mobile Health Application Rating Scale (uMARS)			✓

### ECBI

The ECBI [36] is a 36-item caregiver-report scale measuring frequency (Intensity scale) and perception (Problem scale) of disruptive behavior in children aged 2-16 years. Items are rated on a 7-point scale indicating frequency of the behavior. Scores are presented as T-scores (mean 50, SD 10), with higher scores indicating more frequent behavior problems (Intensity) or greater caregiver perception that behavior is problematic. T-scores of ≥60 are viewed as clinically elevated. Internal consistency in the current sample was high (baseline: α=.90 for Intensity and α=.88 for Problem, follow-up: α=.92 for Intensity and α=.91 for Problem).

### RCB

The RCB [37] is a 30-item scale assessing caregiver attributions for children’s behavior. The RCB includes 7 subscales measuring neurodevelopmental and willful attributions across multiple areas of behavior, including task completion, sensory seeking and avoiding, and dysregulated or disruptive behavior. Items are rated on a 6-point Likert scale ranging from strongly disagree to strongly agree. Higher scores indicate greater agreement with the attribution. Internal consistency across subscales in the current sample was adequate to high (baseline ranging from α=.68 to α=.92, follow-up ranging from α=.67 to α=.95).

### PSOC

The PSOC [38] is a 16-item self-report measure of the caregiver’s sense of parenting efficacy (ie, perceived competence, problem-solving ability, and capability) and satisfaction (ie, the extent to which the individual enjoys the parenting role or experiences parenting frustration and anxiety). The items in the PSOC are answered on a 6-point scale ranging from “strongly disagree” to “strongly agree.” Higher scores on the Efficacy scale indicate lower efficacy, and higher scores on the Satisfaction scale indicate higher satisfaction. Internal consistency in the current sample was high (baseline: α=.80 for satisfaction and α=.80 for Efficacy, follow-up: α=.84 for Satisfaction and α=.80 for Efficacy).

### FMN Questionnaire

The FMN Questionnaire [39] includes 19 items relating to potential needs of caregivers of children with FASD or PAE and assesses the degree to which caregivers perceive these needs have been met. It is based on a measure developed for traumatic brain injury [40] and has been used in several previous studies testing FMF and derivative products [21,24,27,28]. Each need is rated on a 4-point scale (1=not at all met and 4=a great deal met) and is scored by computing the average response across items. Higher scores reflect caregivers’ perceptions of more needs being met. Internal consistency in the current sample was high (baseline α=.90 and follow-up α=.93).

### FASD Questionnaire

The K&A Questionnaire [39] was developed for use in the FMF Program. The version of the K&A used in this study was adapted to emphasize the content most relevant to the FMF Connect app and was informed by response patterns in the larger feasibility trial [21]. It includes 28 core items testing knowledge of FASD, advocacy, and key FMF Program principles. It is scored by calculating the number of items answered correctly. Furthermore, 8 items are rated on a true or false scale, and 20 items are multiple choice. For this study, a total score was computed by summing correct answers. Higher scores indicate higher levels of knowledge of FASD and advocacy. Internal consistency was not calculated for this measure as it measures content mastery targeted by the app versus a unified construct.

### ELS

The ELS was developed for this study to measure caregiver-reported child adaptive functioning. The ELS includes 20 items that are each rated twice by the caregiver, yielding 2 scales, Responsibility and Smoothness. Sample items include “getting ready in the morning,” “making a transition between tasks,” and “calming down when upset.” The Responsibility scale, rated on a 5-point Likert scale, assesses to what degree the child and/or adult is responsible for the behavior. Lower scores reflect that the adult takes full or most responsibility for the behavior, whereas higher scores reflect that the child takes greater responsibility and independence in the behavior. The Smoothness scale, rated on a 5-point Likert scale, assesses how smoothly the behavior goes, ranging from “rarely goes well” to “almost always goes well.” Higher scores reflect greater smoothness. Internal consistency in this study’s sample was high (baseline: α=.92 for Responsibility and α=.88 for Smoothness, follow-up: α=.94 for Responsibility and α=.90 for Smoothness).

### Self-Care Assessment

The Self-care Assessment (SCA) [24,41] was administered at follow-up. Caregivers were asked how their self-care changed over the past 3 months, rated on a 5-point scale (1=a lot less self-care, 2=somewhat less self-care, 3=no change in level of self-care, 4=somewhat more self-care, and 5=a lot more self-care).

### uMARS

The uMARS [42] is a 26-item survey measuring user perception of the quality of mHealth apps. It includes 6 subscales—Engagement, Functionality, Aesthetics, Information, Subjective Quality, and Perceived Impact, and a total App Quality score (summary of first 4 subscales). The uMARS was completed at follow-up only. Caregivers rated the items on a 5-point scale that was specific to each question; higher scores indicate more positive impressions of the app. Internal consistency in this study’s sample was adequate (α=.62 for Engagement, α=.76 for Functionality, α=.82 for Aesthetics, α=.66 for Information, α=.72 for Subjective Quality, α=.93 for Perceived Impact, and α=.93 for App Quality).

### Interventions

#### FMF Connect Intervention

FMF Connect includes cloud infrastructure and an innovative, multilayered mHealth app. It incorporates tailored content for caregivers of children (aged 3‐12 y) with FASD or PAE. The target age group for the FMF Connect app was selected to be the same as the standard FMF Program, from which the app was derived. Content and methods of these programs were designed with caregivers of preschool and school-aged children in mind. The FMF Connect app integrates five main components, (1) Dashboard, (2) Learning Modules, (3) Family Forum, (4) Library, and (5) Notebook (Figure 1). Weekly emails were also sent to support motivational engagement.

App education, derived from the standard FMF Program, is packaged in easily digestible learning modules, including text, exercises, and videos tailored for the child’s age and presenting behaviors. The FMF Connect app includes videos and examples of diverse children across ages 3‐12 years. In the first module, families are asked to provide the age of their child and the main behaviors that concern them. Videos throughout the app are selected from a larger library to be most relevant to the age and primary behavior concerns of the caregiver. The Learning Modules are organized into three levels: (1) Getting Started, (2) Taking Action, and (3) Taking It to the Next Level. The Library provides access to additional optional videos and content that can be downloaded and printed to use in advocacy and skill development. The Notebook is a personalized section that organizes caregivers’ responses to learning activities in a convenient location for later easy access.

The Family Forum serves to (1) engage and facilitate caregivers’ continued usage of the app, (2) support and extend caregivers’ implementation of new knowledge and skills, and (3) provide peer support, encouragement, and understanding of the caregiver’s own needs. Trained peer moderators moderated discussion in the Family Forum to ensure safety. They also provided support and prompt skills or content areas to consider.

The Dashboard displays caregiver progress in Learning Module completion, use of the Family Forum, and daily and weekly ratings inputted by the caregiver. It also includes the “Tip of the Day” feature.

Complementing the app is a Cloud infrastructure to transparently but securely distribute information, including storing and retrieving data, managing notification and messaging, and synchronizing data on all devices. A local datastore protects the mobile app against network failures and connectivity issues. This local datastore is transparently synchronized with the Cloud database so that all querying and security features are constantly available regardless of network connectivity. Updates are automatically pushed so users have the latest version.

Furthermore, 4 minor updates were made to the app during the first 2 months of the trial. These included small wording changes, optimization of the connection with the backend system, adding banners to the Family Forum to easily identify moderators, fixing an issue with the behavior tracker tool affecting some users, adding a new notification management system, and minor bug fixes for performance improvements.

#### Coaching Module

A coaching module was developed within the FMF Connect app to help engage caregivers and support continued use and individualized goal setting within the app. The coaching module was activated for caregivers in the FMF Connect+Coaching group only. This module allowed an assigned coach to exchange text-based messages with caregivers through the FMF Connect app’s notification system. Coaches had access to a dashboard of their participants’ progress and use of the app, which aided in tailoring messages and supporting caregivers in their identified goals. The coach’s interface also provided a secure way to provide text-based messages to the participant within the app.

Coaches were graduate students on the project familiar with FMF Program principles and knowledgeable about FMF Connect content and features. They had training in motivational interviewing (MI), which is a key therapeutic technique used in the FMF Program. Coaches used the spirit and skills of MI to evoke the participant’s own motivation to learn more about FASD and skills taught in FMF Connect. They also aimed to support caregiver goals by helping link them with content and features within FMF Connect.

### Randomization

Randomization was conducted using REDCap’s Randomization package. The allocation sequence was developed by a biostatistician at the University not affiliated with the project to randomly allocate participants equally across the 3 study arms. No stratification or blocking was used. The University Research IT department implemented the allocation table in the REDCap Randomization package, and the research team did not have access to the sequence. Once the study coordinator determined a participant was eligible and baseline measures were complete, the study randomization form would determine the participant’s group status. The coordinator then informed the participant of their group status and sent instructions on next steps (ie, how to install the app or wait time) via email. Participants in the intervention groups were given a username and password and were given instructions on how to install the app from the Apple App Store. The research assistant processing survey data and payments could not access the REDCap Randomization package and was blind to group status.

### Statistical Methods

Data analyses were conducted using SPSS (version 29.0; IBM Corp) and Mplus 8 (Muthén & Muthén). All variables were assessed for outliers, skew, and kurtosis. No extreme outliers or skew or kurtosis were detected. Missing data in SPSS were handled using multiple imputation. Missing data in Mplus used Full Information Maximum Likelihood (FIML).

Demographic variables were analyzed using descriptive statistics in SPSS. Group differences in demographic variables were assessed using 1-way ANOVAs for continuous variables and Pearson chi-square analysis for categorical variables.

Attrition analysis involved analyzing demographic differences at baseline, 6 weeks, and 12 weeks between those who did complete each timepoint and those who did not. For demographics, these differences were conducted using 1-way ANOVAs including all 3 timepoints. Pearson correlations (*r*) were used to detect relationships between baseline variables, demographic variables (caregiver age and child age), and attrition at all timepoints. App usage and perceived app quality were assessed using descriptive statistics and independent samples *t* tests grouped by FMF Connect+Coaching and FMF Connect.

Intervention change analyses were intent-to-treat, including all participants assigned to their respective randomly assigned groups whether or not they completed the FMF Connect program, and involved 2-sided hypothesis testing. Analyses used linear mixed modeling in SPSS for variables collected at the 3 timepoints (RCB, ELS, and PSOC). False discovery rate (FDR) corrections for all *P* values are reported. Simultaneous linear regression was used in Mplus for variables collected at baseline and 12-week follow-up. For regressions, group status and baseline levels were entered in step 1. All regressions were predicting outcomes at 12-week follow-up. FDR correction was used and reported for all regressions. Cohen *d* effect sizes were calculated for each outcome to demonstrate the magnitude of the intervention change. Additionally, simultaneous linear regressions were carried out to assess whether app usage within the FMF group predicted 6-week change scores and 12-week change scores.

Point-biserial correlations between group (FMF Connect vs WLC) and change scores (6-wk timepoint−baseline) were conducted to see if mediation was plausible. For those significant correlations, mediation analyses were conducted in Mplus using change scores as the mediator and the ECBI (child behavior) and PSOC (parenting efficacy or satisfaction) as the outcomes, controlling for baseline levels.

## Results

### Sample Characteristics

Recruitment for this study began on January 6, 2022, with enrollment ending on August 31, 2022. The study was completed on December 31, 2022, after all participants had the opportunity to complete follow-up surveys. Baseline sample demographics can be found in Table 2. No harms or unintended effects were identified for participants.

**Table 2.** Sample demographics at baseline by group.

	FMF^a^ Connect+Coaching (n=43)	FMF Connect (n=43)	WLC^b^ (n=43)	Group difference, *P* value
Age, mean (SD)				
Child	7.3 (2.8)	7.9 (2.6)	8.5 (2.4)	.43
Caregiver	45.2 (7.0)	43.1 (7.6)	43.7 (7.6)	.11
Gender, n (%)				
Child	.31
Boy	21 (49)	28 (65)	24 (56)	
Girl	22 (51)	15 (35)	19 (44)	
Caregiver	.23
Man	0 (0)	2 (5)	3 (7)	
Woman	43 (100)	41 (95)	40 (93)	
Race, n (%)				
Child	.07
White	28 (65)	26 (61)	21 (49)	
Black or African American	6 (14)	0 (0)	8 (19)	
American Indian, Native, or Aboriginal	1 (2)	3 (7)	1 (2)	
Asian	2 (5)	1 (2)	1 (2)	
More than one race	4 (9)	13 (30)	10 (23)	
Unknown	2 (5)	0 (0)	2 (5)	
Caregiver	.42
White	40 (93)	41 (95)	39 (91)	
Black or African American	2 (5)	0 (0)	2 (5)	
American Indian, Native, Aboriginal	0 (0)	0 (0)	0 (0)	
Asian	0 (0)	0 (0)	0 (0)	
More than one race	0 (0)	2 (5)	1 (2)	
Unknown	1 (2)	0 (0)	1 (2)	
Ethnicity, n (%)				
Child	.92
Non-Hispanic or Latine	33 (77)	34 (79)	36 (84)	
Hispanic or Latine	7 (16)	6 (14)	4 (9)	
Unknown	3 (7)	3 (7)	3 (7)	
Caregiver	.23
Non-Hispanic or Latine	41 (95)	43 (100)	40 (93)	
Hispanic or Latine	1 (2)	0 (0)	3 (7)	
Unknown	1 (2)	0 (0)	0 (0)	
Marital status of caregiver, n (%)	.01
Married	32 (74)	36 (84)	33 (77)	
Living with partner	4 (9)	1 (2)	1 (2)	
Separated	0 (0)	0 (0)	3 (7)	
Divorced	1 (2)	6 (14)	4 (9)	
Never married	6 (14)	0 (0)	2 (5)	
Education of caregiver, n (%)	.28
Did not complete high school	1 (2)	0 (0)	1 (2)	
High school degree	1 (2)	0 (0)	1 (2)	
Partial college	4 (9)	7 (16)	2 (5)	
Undergraduate degree	16 (37)	22 (51)	13 (30)	
Partial graduate	5 (12)	2 (5)	6 (14)	
Graduate degree	16 (37)	12 (30)	20 (47)	
Relationship to child, n (%)	.21
Biological mother	1 (2)	0 (0)	0 (0)	
Biological father	0 (0)	0 (0)	2 (5)	
Grandparent	2 (5)	3 (7)	1 (2)	
Other relative	1 (2)	2 (5)	3 (7)	
Adoptive parent	37 (86)	39 (91)	35 (81)	
Foster parent	5 (12)	1 (2)	3 (7)	
Stepparent	2 (5)	0 (0)	1 (2)	
Income (in thousands; US $), mean (SD)	118.2 (76.0)	110.9 (66.2)	108.8 (65.0)	.80
Household structure, mean (SD)				
People in home	4.9 (1.7)	5.0 (1.5)	5.0 (1.6)	.99
Children in home	3.1 (1.6)	3.1 (1.5)	3.1 (1.6)	.99

a FMF: Families Moving Forward.

b WLC: waitlist control group.

Baseline correlations by group can be found in Multimedia Appendix 1.

### Demographic Group Differences

There were no significant differences between the FMF Connect+Coaching, FMF Connect, and WLC groups on any continuous variables (age, income, and household structure), nor were there significant group differences among the majority of categorical variables (gender, race, ethnicity, and relationship to child). There was a significant group difference on variables characterizing caregiver marital status. However, given the small size of marital status subgroups, this variable was not controlled for in further analyses. Given the absence of group differences, no other demographic factors were controlled for.

### Attrition Analysis

**Figure 2.**
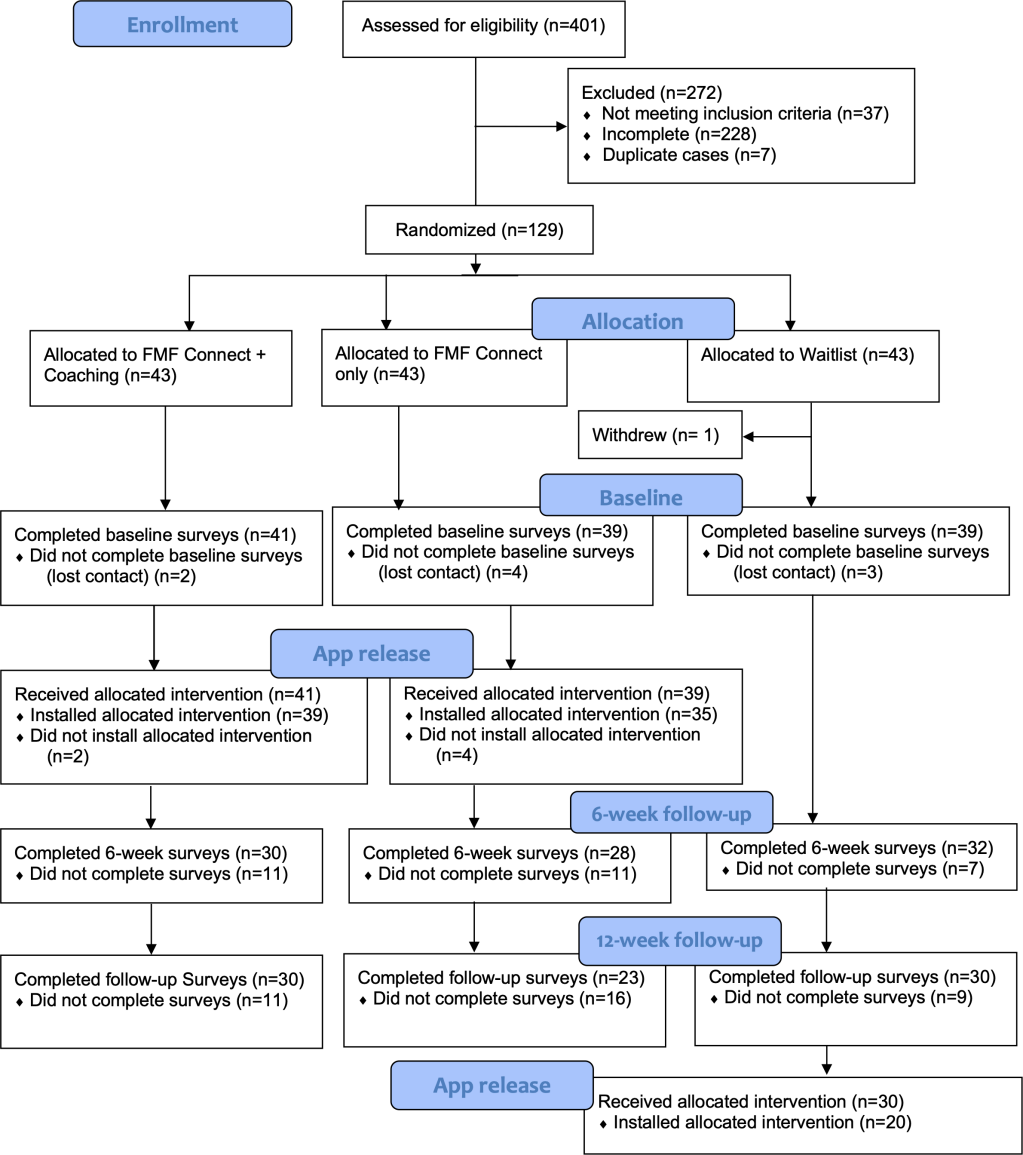
Study CONSORT (Consolidated Standards of Reporting Trials) diagram for the FMF Connect mobile health intervention randomized controlled trial. FMF: Families Moving Forward.

The trial CONSORT (Consolidated Standards of Reporting Trials) diagram is presented in Figure 2. There were no significant differences in demographic characteristics (caregiver’s gender, caregiver’s race or ethnicity, caregiver’s education level, comfort with technology, knowledge of FASD, income, child’s gender, child’s race or ethnicity, and caregiver type) between those who did and did not complete data collection at the 6-week or 12-week timepoints. Caregiver’s age and child’s age were not correlated with attrition. *t*-tests resulted in no significant difference in attrition by number of children in the home.

Differential attrition was also assessed for in baseline levels of functioning across groups. Greater attrition was identified among caregivers who, at baseline, reported lower child responsibility for daily tasks on the ELS for completion at 6 weeks (*t*_117_=−2.85; *P*=.003) and 12 weeks (*t*_117_=−2.69; *P*=.004). No other baseline scores were found to correlate significantly to attrition.

### Perceived App Quality

Table 3 provides means, SDs, and score ranges on the uMARS measure of perceived app quality by users in the 2 intervention groups with data at 12 weeks. Consistent with a past feasibility trial [21], users reported high perceived quality and satisfaction with the app, with nearly all scores averaging at or above 4 (maximum 5). The quality of information provided remains the highest rated domain across groups, consistent with the goal of the FMF Connect app. This scale alone was significantly different across groups. The FMF Connect+Coaching group reported slightly lower mean ratings on quality of information than did the FMF Connect app group.

**Table 3.** App quality assessment by intervention group at 12 weeks.

	FMF Connect+Coaching (n=29^a^) mean (SD; range)	FMF Connect (n=22^a^) mean (SD; range)	Group differences, *P* value
Engagement	3.8 (0.5; 3.0-4.8)	3.9 (0.3; 3.2-4.4)	.49
Functionality	4.2 (0.5; 3.3-5.0)	4.2 (0.48; 3.0-5.0)	.53
Aesthetics	4.1 (0.6; 3.0-5.0)	4.2 (0.6; 2.7-5.0)	.59
Information	4.5 (0.4; 3.8-5.0)	4.7 (0.3; 4.3-5.0)	.03
App Quality	4.1 (0.4; 3.4-5.0)	4.2 (0.3; 3.6-4.8)	.26
Subjective Quality	4.1 (0.6; 2.8-5.0)	4.1 (0.8; 2.5-5.0)	.99
Perceived Impact	4.0 (0.8; 1.3-5.0)	4.3 (0.6; 3.0-5.0)	.26

a Number corresponds to all participants with data on the respective measures at that time point. Participants are included in their assigned group irrespective of app usage patterns. Some participants used the app but did not complete the uMARS at 12-week surveys.

### App Usage

App usage in the FMF Connect groups was measured through number of app openings, number of modules completed, number of fact sheet openings, and number of videos watched. Table 4 provides means, SDs, and ranges for participants in each of the intervention groups who registered usage in the app (which is higher than the number who completed follow-up surveys).

**Table 4.** App usage by intervention group.

	FMF Connect+Coaching (n=39^a^), mean (SD; range)	FMF Connect (n=35^a^), mean (SD; range)	Group differences, *P* values
App openings	27.18 (26.53; 2-111)	42.23 (143.60; 1-861^b^)	.52
Learning Module sections opened (out of 131 maximum^c^)	67.54 (54.46; 0-165)	55 (47.47; 0-175)	.30
Learning Module sections completed	61.23 (50.52; 0-148)	49.14 (42.98; 0-138)	.27
Fact sheets opened	7.69 (11.03; 0-50)	7.37 (12.15; 0-65)	.91
Videos started	1.46 (6.78; 0-41)	2.8 (10.21; 0-53)	.51
Videos completed	1.15 (5.95; 0-37)	2.31 (9.26; 0-49)	.52

a Number corresponds to all participants with data on the respective measures at that time point. Participants are included in their assigned group irrespective of app usage patterns.

b The participant with 861 app openings in the FMF Connect group was an extreme outlier. When this outlier is removed, app opening descriptives are as follows: 18.15 (18.28), 1-75. Group differences are still non-significant (*P*=.10).

c While there were 131 maximum learning module sections, modules could have been initiated or completed more than once resulting in a range larger than 131.

For those assigned to coaching, participants messaged their coach an average of 2.95 (SD 2.72) times. The number of messages sent from the participant to their coach ranged from 0 to 10, with 10 participants never using the coaching feature. Coaches messaged participants an average of 6.55 (SD 3.61) times. The number of messages sent from coaches to the participant ranged from 1 to 17.

App usage was not significantly different between the FMF Connect+Coaching and FMF Connect only groups on any of the usage measures, which suggests coaching did not significantly impact app engagement as hypothesized. Pearson *r* correlations were used to see if demographic and baseline data were related to app usage across FMF Connect groups. In terms of demographic factors, older caregivers opened the app (*r*=0.39, *P*<.001) and fact sheets (*r*=0.31, *P*=.009) more than younger caregivers. Caregivers who had less comfort with technology opened fewer PDFs in the Library (*r*=−0.23, *P*=.048). No other demographic factors were associated with app usage.

In terms of baseline measures, caregivers with lower parenting efficacy opened the app (*r*=−0.31, *P*=.007) and fact sheets (*r*=−0.30, *P*=.01) more. Caregivers with fewer family needs met opened the app (*r*=−0.25, *P*=.03) and fact sheets (*r*=−0.25, *P*=.03) more than families with greater needs met at baseline. Caregivers with lower baseline FASD knowledge (K&A) finished fewer Learning Modules (*r*=0.24, *P*=.04). Caregivers who rated everyday life tasks as less smooth (ELS) opened the app (*r*=−0.27, *P*=.02) and fact sheets (*r*=−0.27, *P*=.02) more. Caregivers with higher sensory seeking attributions (RCB) opened the app (*r*=−0.26, *P*=.03) and fact sheets (*r*=−0.26, *P*=.03) less often. Those with higher ability-based task attributions watched fewer videos (*r*=−0.23, *P*=.046). In summary, these score patterns suggest that caregivers reporting greater difficulties (eg, less smooth daily tasks, lower efficacy, and fewer needs met) generally engaged with the app more than those with relatively fewer difficulties.

### Intervention Change

The FMF Connect+Coaching and FMF Connect groups were aggregated for analyses examining intervention change, given minimal group differences across demographic characteristics, baseline and follow-up measures, and app usage. Analyses conducted with 3 separate groups, as documented on clinicaltrials.gov, can be found in Multimedia Appendix 2. These analyses did not use intent-to-treat or multiple imputation methodologies; therefore, they should be interpreted with caution.

Table 5 reports linear mixed models for all intervention outcomes with 3 time points (RCB, PSOC, and ELS). Pairwise comparisons for significant models are further discussed in this study.

**Table 5.** Linear mixed modeling for intervention change.

Measure	F test (*df*)	*P* value	FDR^a^
Primary outcomes			
RCB^b^ Sensation Avoiding attributions	0.65 (2)	.52	0.60
RCB Sensation Seeking attributions	0.61 (2)	.55	0.89
RCB Task Willful attributions	4.60 (2)	.02	0.14
RCB Task Ability attributions	0.78 (2)	.46	0.89
RCB Disruptive attributions	2.54 (2)	.08	0.46
RCB Emotion Seeking attributions	0.04 (2)	.96	0.96
RCB Dysregulated attributions	1.70 (2)	.19	0.52
PSOC^c^ Parenting Efficacy	0.38 (2)	.68	0.82
PSOC Parenting Satisfaction	2.08 (2)	.13	0.48
Secondary outcomes			
ELS^d^ child or caregiver Responsibility of everyday tasks	0.61 (2)	.54	0.75
ELS Smoothness of everyday tasks	0.30 (2)	.74	0.82

a FDR: false discovery rate correction.

b RCB: Reasons for Child Behavior.

c PSOC: Parenting Self-Efficacy and Satisfaction.

d ELS: Everyday Life Scale.

### RCB (Primary Outcome)

**Figure 3.**
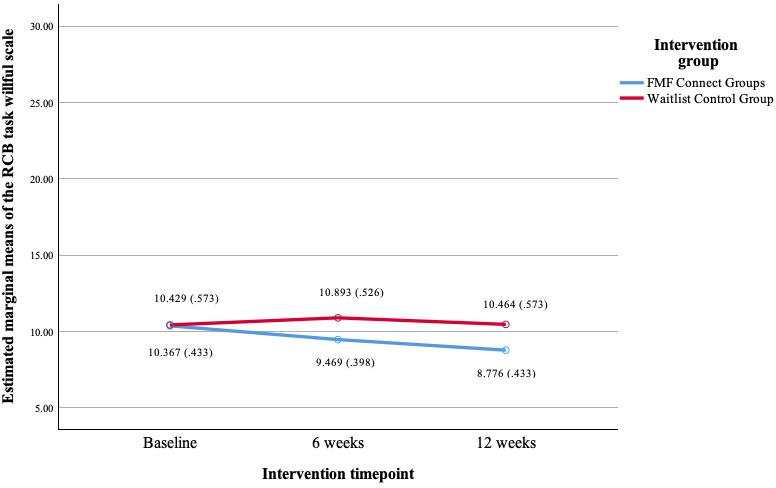
Intervention group differences by timepoint on the Reasons for Child Behavior Scale Task Willful subscale (FMF Connect vs waitlist control). FMF: Families Moving Forward; RCB: Reasons for Child Behavior Scale.

There was a significant group×timepoint interaction on the RCB Task Willful measure (*F*_2_=4.60, *P*=.01; Figure 3). Pairwise comparisons indicate significant group differences at the 12-week timepoint (mean difference=0.86, *P*=.002), with the FMF Connect group demonstrating lower endorsement of task willful attributions (mean 9.63, SE 0.30) compared with the control group (mean 10.13, SE 0.43). Interactions are no longer statistically significant when applying FDR correction.

No differences were detected for parenting efficacy and satisfaction or child adaptive behavior.

Linear regression analyses were conducted for all outcome measures collected at baseline and 12-week follow-up timepoints only (ECBI, FNM, K&A, and SCA). Baseline and FMF Connect group status were entered into the regression model simultaneously. The dependent variable entered was the measure at the 12-week follow-up to determine treatment effects (Table 6). Covariates were not included in analyses as there were minimal correlations or group differences on any of the demographic variables assessed (Table 2). Significant findings are summarized further in this study. FDR corrections for all *P* values are reported.

**Table 6.** Linear regression and Cohen *d* effect sizes for intervention change.

Measure	B (SE)	b	*P* value	FDR^a^	Cohen *d* (95% CI)
Primary outcomes					
ECBI^b^ Child Behavior Intensity					−0.22 (−0.69 to 0.23)
Baseline	0.81 (0.07)	0.80	<.001	<0.001	
Group	−1.18 (1.24)	−0.06	.33	0.33	
ECBI Child Behavior Problem					−0.35 (−0.80 to 0.11)
Baseline	0.80 (0.10)	0.67	<.001	<0.001	
Group	−2.82 (1.87)	−0.12	.13	0.14	
Family Needs Met					0.49 (−0.02 to 0.94)
Baseline	0.45 (0.12)	0.38	<.001	<0.001	
Group	4.91 (2.38)	0.21	.04	0.06	
K&A^c^ FASD^d^ Knowledge					0.47 (0.02 to 0.93)
Baseline	.64 (.10)	0.57	<.001	<0.001	
Group	1.18 (0.57)	0.18	.04	0.06	
Secondary outcomes					
SCA^e^ Caregiver Self-Care					0.46 (0.00 to 0.91)
Baseline	0.64 (0.10)	0.57	<.001	<0.001	
Group	0.93 (0.47)	0.17	.048	0.07	

a FDR: false discovery rate correction.

b ECBI: Eyberg Child Behavior Inventory.

c K&A: Knowledge & Advocacy Questionnaire.

d FASD: fetal alcohol spectrum disorders.

e SCA: Self-Care Assessment.

### FMF (Primary Outcome)

Group status (FMF Connect: n=80 and control: n=39) significantly predicted the FNM summary score at 12 weeks (*b*=0.21, *P*=.04), when accounting for scores at baseline and accounting for the interaction. The FMF Connect group showed significantly higher scores on the FNM post intervention (mean 47.56, SD 11.06) compared with WLC (mean 42.30, SD 11.29), indicating more family needs met. This prediction approaches significance when adjusting for multiple comparisons (*P*=.06).

### K&A (Primary Outcome)

Group status (FMF Connect: n=80 and control: n=39) significantly predicted the K&A summary score at 12 weeks (*b*=0.18, *P*=.04), when accounting for scores at baseline. The FMF Connect group showed significantly higher scores on the K&A post intervention (mean 21.28, SD 3.28) compared with the WLC group (mean 20.33, SD 2.69), meaning they acquired greater knowledge about FASD, advocating for their child, and core FMF Program concepts. This prediction approaches significance when adjusting for multiple comparisons (*P*=.06).

### Self-Care (Secondary Outcome)

Group status (FMF Connect: n=80 and control: n=39) significantly predicted the SCA score at 12 weeks (*b*=0.17, *P*=.048), when accounting for scores at baseline. The FMF Connect group showed significantly higher scores on the SCA post intervention (mean 5.38, SD 2.46) compared with the WLC group (mean 4.401, SD 2.59), meaning they acquired more self-care skills compared with waitlist controls. This prediction approaches significance when adjusting for multiple comparisons (*P*=.07).

Group status did not predict child behavior (ECBI) at 12 weeks when controlling for scores at baseline.

### App Usage and Intervention Change (Secondary Outcome)

Learning module completion and app opening were entered into a simultaneous regression model to predict change scores at the 6-week and 12-week timepoints (Multimedia Appendix 3). Learning module completion (*b*=−0.20, *P*=.02) and app openings (*b*=0.79, *P*=.02) had an effect on PSOC Efficacy change scores over 12 weeks, in that those who completed more learning modules and opened the app more had greater levels of parenting efficacy after 12 weeks. Caregivers who opened the app more showed more improvement on RCB Emotion Seeking at the 12-week timepoint (*b*=0.76, *P*=.03) and PSOC Efficacy at the 6-week timepoint (*b*=0.35, *P*=.002).

### Mediation Hypotheses

**Figure 4.**
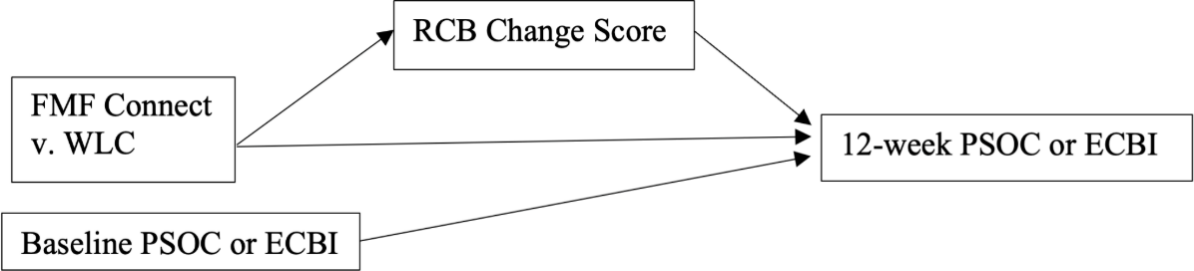
Mediation model testing whether caregiver attributions at 6 weeks (measured by the Reasons for Child Behavior scale) mediate the intervention effect of receiving the Families Moving Forward Connect app versus the waitlist control on parenting self-efficacy or satisfaction at 12 weeks (measured by the Parenting Sense of Competence scale). ECBI: Eyberg Child Behavior Inventory; FMF: Families Moving Forward; PSOC: Parenting Sense of Competence scale; RCB: Reasons for Child Behavior scale; WLC: waitlist control.

Point-biserial correlations between group (FMF Connect vs WLC) and change scores (6-week timepoint−baseline) were conducted to see if mediation was plausible. Group status was correlated with 6-week change scores on the RCB Disruptive Behavior (*r*=−0.24, *P*=.03) and RCB Task Willful (*r*=−0.26, *P*=.02) scales. Mediation analyses were conducted in Mplus using the model illustrated (Figure 4) looking at both the RCB Disruptive Behavior and Task Willful scores as the mediator and the ECBI and PSOC as the outcome. No significant mediation was found.

## Discussion

### Primary Findings

Families raising children with FASD experience poor access to evidence-based care in their communities given multiple systems-level barriers. These include limited professional training in FASD, long waitlists and provider shortages, and pervasive stigma [9,12,43]. Complementing efforts to build workforce capacity, this study tested the efficacy of FMF Connect, a first of its kind—a caregiver self-directed mHealth intervention. Results from this RCT provide initial support for the efficacy of the FMF Connect app for caregivers raising preschool and school-aged children with FASD or PAE. Relative to the waitlist group, groups that received the FMF Connect app had greater increases in FASD knowledge, reductions in willful attributions of child behavior, improved self-care practices, and more family needs met. These changes in caregiver outcomes are notable given the low intensity, self-directed nature of the intervention. Contrary to hypotheses, no significant group differences were found for child outcomes assessed. Consistent with our previous feasibility trial [21], caregivers gave high ratings for app quality, with the quality of information rated highest among indicators. App usage was relatively greater for caregivers reporting more difficulties with parenting efficacy, completing daily tasks with their children, and meeting family needs. Higher app usage was also related to greater changes in parenting efficacy scores across the 12-week intervention period.

The systematic development and testing of the FMF Connect app is particularly notable given that a 2024 review of parenting apps found that none of the available apps in app stores had been empirically tested [13]. The FMF Connect app is also part of a growing continuum of products, all derived from the standard FMF Program to meet the varying needs of children and youth of different ages, the families who care for them, and different settings in which they are treated. Similar to the standard specialist-led FMF Program, the FMF Connect app is related to important changes in caregiver and family functioning [24,27,28]. The magnitude of effect sizes for the FMF Connect app (small-medium) is generally smaller than the standard FMF Program (many medium-large), which is not surprising given the difference in duration (12 wk vs 9 mo) and format (self-directed vs specialist implemented) of the interventions. Dosage of content is also much more variable with the FMF Connect app (intervention groups averaging 56 of 131 core Learning Module sections; range 0‐148, including repeated content); past trials with the FMF Program have had nearly all families complete the full program. Across studies, the FMF Program and FMF Connect app result in consistent improvements in FASD knowledge and family needs met. Changes in caregiver attributions of child behavior have also been found across studies, although measurement approaches have been variable. Interestingly, studies testing the standard FMF Program have found medium to large effects in parenting efficacy, whereas this study did not find significant group differences after access to the FMF Connect app. However, higher levels of app usage did relate to greater change in parenting efficacy scores, suggesting greater dosage of content is important for this outcome. Caregivers across studies have reported a high level of satisfaction with both interventions. While the magnitude of effects for the FMF Connect app is more modest, the potential for scalability creates the possibility of a broad public health impact. This impact and reach could potentially be leveraged as additional products in the FMF continuum of care are developed, continuing to increase accessibility and capacity to provide evidence-based care.

### Unsupported Hypotheses

In contrast to our hypothesis, text-based coaching did not result in improved app usage or outcomes for families. Across a broad range of mHealth apps designed to manage health conditions in adults, a positive correlation has been found between app engagement and the level of personal support provided [44]. However, there is a high level of heterogeneity in methods and outcomes for mHealth interventions using coaching, and many studies did not directly assess the effect of coaching on outcomes [45]. Another possible explanation for the pattern of findings in this study is that user engagement in coaching was lower than anticipated. Study team observations noted that a few users reported difficulty finding or navigating the coaching messaging component. It is also possible that the users turned off app notifications that would have alerted them to new messages or were not interested in this feature. A future direction could explore whether peer coaches (vs graduate students) increase engagement.

We also predicted that more app usage would relate to greater improvement in outcomes. However, this hypothesis was not generally supported; usage did not correlate with greater effects for the majority of outcomes, with the exception of parenting efficacy noted above. Possibly, there is a threshold effect after accessing a certain amount (or type) of content. Alternatively, change may be related to more individualized patterns of usage or needs based on caregiver and family circumstances. Future larger-scale investigations may allow for greater power and ability to use machine learning techniques to explore usage patterns in more nuanced ways.

Finally, based on the theorized logic model for the FMF Program, this study hypothesized that caregiver attributions of behavior would mediate changes in caregiver efficacy and satisfaction and child behavior outcomes. Results of this study did not support this hypothesis. While the intervention group was correlated with 6-week change on 2 scales, mediation was not significant. In addition, the app was not associated with change in parenting efficacy, satisfaction, or child behavior over the 12-week evaluation period. Given intervention effects are seen on these outcomes in the longer duration follow-ups for the specialist-led FMF program [24,27,28], it is possible that more intensive intervention and/or follow-up is needed for these outcomes.

### Strengths and Limitations

Results from this study should be interpreted in the context of several notable strengths and limitations. First, this study empirically tested one of the first stand-alone, self-directed mHealth interventions targeting parenting. Although the FMF Connect app is tailored to caregivers raising children with FASD, findings from this study have broader relevance for parenting programs and use of mHealth interventions more generally. Much of the content and form of FMF Connect could be adapted and trialed with other related populations. The FMF Connect app was rigorously developed and tested using a systematic user-centered design approach [21-23]. The app was derived from an evidence-based intervention with a strong theoretical foundation and adapted for self-directed administration on a mobile platform. Our approach is consistent with best practices in mental health app development and broader principles of user-centered design [20,46].

The generalizability of study findings may well be impacted by characteristics of study participants who enrolled in the study and other design features. Although careful attention was paid to participant reach in previous feasibility trials [21] and efforts were made to recruit a diverse participant pool, enrolled participants were largely White, relatively wealthy, adoptive mothers. Caregiver education and income were higher than national averages. Our study was also limited to participants with Apple iOS devices, which may have influenced patterns of participant demographics. For example, commercial survey data indicate iOS users tend to have higher average income, longer duration of smartphone use per day, and be younger in age than Android users [47]. The decision to limit the study to iOS devices was made given poorer stability of our Android prototypes across user devices in previous feasibility studies [21,22]. This decision likely eliminated about 40% of potential participants based on rates of Android users in our past studies and current market share in the United States [47]. Expanding the app to the Android platform and further engagement or co-design work with demographic groups not well-represented in this study are important future steps to address this limitation. Since this trial, we have subsequently rebuilt the app on Flutter (Google), which allows seamless development and refinement across iOS, Android, and web platforms. We are also in the process of commercializing the app for broad distribution. Some families may not be interested in participating in research due to barriers or mistrust but might be more likely to download the app from the app store and use it outside of the research context. It is possible these steps will increase the diversity of users. Additionally, targeted outreach and collaboration with community organizations serving underrepresented populations could help address this generalizability gap.

As with most behavioral intervention studies, participants were aware of their treatment condition, which could have potentially biased follow-up responses. FMF Connect targeted multiple child and family outcomes. As a result, multiple analyses were run to assess the range of possible intervention effects targeted. The number of analyses may have increased the likelihood of false positive intervention effects, as suggested by FDR corrections.

### Conclusions and Future Directions

This study provided initial evidence for the efficacy of the FMF Connect app for targeted caregiver outcomes, with effect sizes in the small to medium range. As an mHealth app, the FMF Connect intervention has potential for scalability and accessibility. Even with modest effect sizes, this potential for scalability could result in a sizeable public health impact, especially for families who may have difficulty accessing evidence-based resources in their communities or have other barriers to care. The FMF Connect app is not intended to replace diagnostic and treatment services delivered by providers. Yet, given the severe lack of FASD-informed providers in the United States, this resource can help provide families with easy access to evidence-based information and ideas for how to access and advocate for services in their communities. Beyond that, through the Family Forum, the app can provide connections with other families with similar experiences, providing peer support.

Building on this work, our team is developing and testing a new program called FMF Connect Pro to further expand the continuum of care for families based on the FMF Program. This effort aims to train mental health providers in how to screen for PAE, diagnose FASD (using Neurobehavioral Disorder Associated with Prenatal Alcohol Exposure in the *DSM-5* [*Diagnostic and Statistical Manual of Mental Disorders* {Fifth Edition}] [3]), and support families using the FMF Connect app within their clinical practice. This middle tier between the self-directed FMF Connect app and the standard specialist-led FMF Program has the potential to increase identification of FASD in settings with increased prevalence [48,49] and enhance access to FASD-informed treatment for families. It could also provide clinicians with easy-to-use tools and a lower training investment option to gain competencies with this population across a range of mental health settings. It is also possible that caregiver-professional relationships may increase engagement and use with families, especially those less likely to use technology on their own. Engagement with FMF Connect Pro could also increase future provider uptake of the standard specialist-led FMF Program, which may often be the best fit for higher-acuity children and families.

## Supplementary material

10.2196/73647 Multimedia Appendix 1 Baseline variable correlations by group (FMF Connect/Waitlist).

10.2196/73647 Multimedia Appendix 2 FMF Connect app usage predicting intervention change scores.

10.2196/73647 Multimedia Appendix 3 Clinical Trials registration three-group (FMFa Connect vs FMF Connect+Coaching vs Control) ANOVA analyses (baseline and 12-week timepoints).

10.2196/73647 Checklist 1 CONSORT checklist.

## Abbreviations

AWS: Amazon Web Services
CIFASD: Collaborative Initiative on Fetal Alcohol Spectrum Disorders
CONSORT: Consolidated Standards of Reporting Trials
*DSM-5*: *Diagnostic and Statistical Manual of Mental Disorders* (Fifth Edition)
ECBI: Eyberg Child Behavior Inventory
ELS: Everyday Life Scale
FASD: fetal alcohol spectrum disorders
FDR: false discovery rate
FIML: Full Information Maximum Likelihood
FMF: Families Moving Forward
FNM: Family Needs Questionnaire
K&A: Knowledge & Advocacy Questionnaire
mHealth: mobile health
MI: motivational interviewing
PAE: prenatal alcohol exposure
PSOC: Parenting Sense of Competence Scale
RCB: Reasons for Children’s Behavior Scale
RCT: randomized controlled trial
REDCap: Research Electronic Data Capture
SCA: Self-care Assessment
uMARS: User Version of the Mobile Health Application Rating Scale
WLC: waitlist control
