# Supplementary material for: Families Moving Forward Connect mHealth Intervention for Caregivers of Children With Fetal Alcohol Spectrum Disorders: Randomized Controlled Trial
Source: JMIR Mhealth Uhealth. 2026 Mar 20;14:e73647. doi: 10.2196/73647 (PMC13004560; doi:10.2196/73647)
Supplement: Multimedia Appendix 1 [file mhealth-v14-e73647-s001.docx]

Multimedia Appendix 1. Baseline Correlations by Group (FMF Connect/WLC *^a^*)

|  | **1.^c^** | **2.^d^** | **3.^e^** | **4.^f^** | **5.^g^** | **6.^h^** | **7.^i^** | **8.^j^** | **9.^k^** | **10.^l^** | **11.^m^** | **12.^n^** | **13.^o^** | **14.^p^** | **15.^q^** |
| --- | --- | --- | --- | --- | --- | --- | --- | --- | --- | --- | --- | --- | --- | --- | --- |
|  | -/- | .035/  -.032 | .029/  -.179 | -.044/  .018 | -.086/  -.203 | -.054/  -.235 | -.220/  -.049 | .018/  .161 | .251*/.150 | .094/  -.073 | -.068/  .162 | .255*/.354* | .380**/.344* | -.244*  /-.214 | -.353*  /-.067 |
|  |  | -/- | .208/  .384^*b^ | .200/  -.232 | -.105/  -.009 | .331^*^/  .316* | -.207/  .175 | .221^*/^  .448* | -.109/  -.294 | -.167/  -.238 | .092/  .371* | -.022/  -.112 | .003/  -.158 | .071/  .371* | .148/  .488* |
|  |  |  | -/- | .290^*/^  .073 | .097/  .090 | .170/  .568* | -.187/  .059 | .275^* /^  -.058 | .114/  -.273 | .102/  -.065 | .048/  .161 | -.044/  .001 | .077/  .048 | .108/  .357* | .167/  .393* |
|  |  |  |  | -/- | .094/  -.017 | .261^*/^  .072 | -.189/  -.035 | .413^*/^  .028 | -.161/  -.018 | -.120/  .069 | -.026/  .029 | -.052/  .023 | -.070/  .027 | .224^*/^  -.089 | .370^*/^  -.034 |
|  |  |  |  |  | -/- | .048/  .080 | .522^*/^  .507* | -.055/  -.112 | -.248^*/^  -.365^*^ | .163/  .004 | -.254^*^/  .001 | -.333^*/^  -.387^*^ | -.488^*/^  -.492^*^ | .550^*/^  .264 | .334^*/^  .143 |
|  |  |  |  |  |  | -/- | -.043/  .069 | .193/  -.161 | -.204/  -.272 | -.280^*^/  -.186 | .036/  .128 | -.133/  -.038 | -.215/  -.090 | .150/  .300 | .302^*/^  .317^*^ |
|  |  |  |  |  |  |  | -/- | -.010/  .194 | -.398^*/^  -.532^*^ | -.163/ -.145 | -.239^*/^  .187 | -.330^*/^  -.447* | -.515^*/^  -.501^*^ | .492^*/^  .537^*^ | .387^*/^  .580^*^ |
|  |  |  |  |  |  |  |  | -/- | -.246^*/^  -.346^*^ | -.355^*/^  -.260 | -.158/  .230 | -.048/  .029 | -.132/  -.086 | .214/  .358^*^ | .396^*/^  .563^*^ |
|  |  |  |  |  |  |  |  |  | -/- | .639^*/^  .570^*^ | .276^*/^  -.255 | .368*/  .200 | .366^*/^  .326^*^ | -.518^*/^  -.690^*^ | -.684^*/^  -.714^*^ |
|  |  |  |  |  |  |  |  |  |  | -/- | .346^*/^  -.126 | .093/  -.114 | .134/  -.012 | -.143/  -.281 | -.355^*/^  -.365^*^ |
|  |  |  |  |  |  |  |  |  |  |  | -/- | -.112/  .050 | .044/  -.098 | -.170/  .254 | -.209/  .318^*^ |
|  |  |  |  |  |  |  |  |  |  |  |  | -/- | .608^*/^  .568^*^ | -.376^*/^  -.286 | -.250^*/^  -.217 |
|  |  |  |  |  |  |  |  |  |  |  |  |  | -/- | -.441^*/^  -.506^*^ | -.425^*/^  -.289 |
|  |  |  |  |  |  |  |  |  |  |  |  |  |  | -/- | .639^*/^  .761^**^ |
|  |  |  |  |  |  |  |  |  |  |  |  |  |  |  | -/- |

*^a^*Families Moving Forward Connect group / Waitlist control group

^b^* = significant *p* < .05

^c^1= Family Needs Met scale (Primary Outcome)

^d^2 = Reasons for Child Behavior (RCB) Sensory Avoidant scale (Primary Outcome)

^e^3= RCB Sensory Seeking scale (Primary Outcome)

^f^4 = RCB Task Ability scale (Primary Outcome)

^g^5 = RCB Task Willful scale (Primary Outcome)

^h^6 = RCB Emotion Seeking scale (Primary Outcome)

^i^7 = RCB Disruptive scale (Primary Outcome)

^j^8 = RCB Dysregulated scale (Primary Outcome)

^k^9 = Everyday Life Scale (ELS) Smooth scale (Secondary Outcome)

^l^10 = ELS Responsibility scale (Secondary Outcome)

^m^11 = FASD Knowledge and Advocacy scale (Primary Outcome)

^n^12 = Parenting Sense of Competence (PSOC) Efficacy scale (Primary Outcome)

^o^13 = PSOC Satisfaction scale (Primary Outcome)

^p^4 = Eyberg Child Behavior Inventory (ECBI) Problem scale (Primary Outcome)

^q^15 = ECBI Intensity scale (Primary Outcome)
