# Supplementary material for: Families Moving Forward Connect mHealth Intervention for Caregivers of Children With Fetal Alcohol Spectrum Disorders: Randomized Controlled Trial
Source: JMIR Mhealth Uhealth. 2026 Mar 20;14:e73647. doi: 10.2196/73647 (PMC13004560; doi:10.2196/73647)
Supplement: Multimedia Appendix 2 [file mhealth-v14-e73647-s002.docx]

Multimedia Appendix 2. Clinical Trials Registration Three-Group (FMF^a^ Connect v. FMF Connect+Coaching v. Control) ANOVA Analyses (Baseline and 12-week Timepoints)

| Measure | F | *p*-value |
| --- | --- | --- |
| **ECBI^b^ Intensity** | 0.511 | .602 |
| **RCB^c^ Sensory Avoiding** | 1.245 | .294 |
| **RCB Sensory Seeking** | 2.585 | .039 |
| **RCB Task Willful** | 2.723 | .032 |
| **RCB Task Ability** | 0.588 | .672 |
| **RCB Disruptive Behavior** | 2.612 | .038 |
| **RCB Emotional Support** | 0.196 | .940 |
| **RCB Dysregulated Behavior** | 0.590 | .671 |
| **PSOC^d^ Efficacy** | 0.896 | .468 |
| **PSOC Satisfaction** | 3.420 | .010 |
| **Family Needs Met** | 1.384 | .257 |
| **K&A^e^** | 1.686 | .192 |
| **Self-Care & Advocacy** | 0.651 | .524 |
| **App Quality** | 1.320 | .255 |

^a^FMF = Families Moving Forward

^b^ECBI = Eyberg Child Behavior Inventory

^c^RCB = Reasons for Child Behavior scale

^d^PSOC = Parenting Sense of Competency Scale

^e^K&A = FASD Knowledge and Advocacy Scale
