# Supplementary material for: Families Moving Forward Connect mHealth Intervention for Caregivers of Children With Fetal Alcohol Spectrum Disorders: Randomized Controlled Trial
Source: JMIR Mhealth Uhealth. 2026 Mar 20;14:e73647. doi: 10.2196/73647 (PMC13004560; doi:10.2196/73647)
Supplement: Multimedia Appendix 3 [file mhealth-v14-e73647-s003.docx]

Multimedia Appendix 3. FMF Connect App Usage Predicting Intervention Change Scores

| Measure | B (SE^a^) | Beta | *p*-value |
| --- | --- | --- | --- |
| Primary Outcomes |  |  |  |
| **ECBI^b^ Intensity 12-week change**  App Openings  Module Completion | -.001 (.035)  .000 (.017) | -.114  .004 | .843  .979 |
| **ECBI Problem 12-week change**  App Openings  Module Completion | -.062 (.051)  .005 (.025) | -.578  .023 | .225  .837 |
| **RCB^c^ Dysregulation 6-week change**  App Openings  Module Completion | .001 (.003)  -.002 (.006) | .050  -.040 | .682  .746 |
| **RCB Dysregulation 12-week change**  App Openings  Module Completion | -.001 (.012)  .000 (.007) | -.052  -.003 | .906  .980 |
| **RCB Disruptive 6-week change**  App Openings  Module Completion | .000 (.005)  -.017 (.011) | .009  -.186 | .938  .125 |
| **RCB Disruptive 12-week change**  App Openings  Module Completion | -.027 (.019)  -.002 (.012) | -.517  -.017 | .150  .878 |
| **RCB Emotion Seeking 6-week change**  App Openings  Module Completion | .000 (.001)  .000 (.001) | -.104  .026 | .391  .835 |
| **RCB Emotion Seeking 12-week change**  App Openings  Module Completion | .034 (.016)  -.004 (.008) | .761  -.049 | .034  .582 |
| **RCB Task Willful 6-week change**  App Openings  Module Completion | .003 (.003)  .000 (.006) | .106  .005 | .380  .969 |
| **RCB Task Willful 12-week change**  App Openings  Module Completion | -.001 (.013)  .004 (.007) | -.058  .083 | .916  .536 |
| **RCB Task Ability 6-week change**  App Openings  Module Completion | .002 (.004)  .008 (.009) | .065  .100 | .588  .417 |
| **RCB Task Ability 12-week change**  App Openings  Module Completion | .021 (.020)  .004 (.011) | .461  .040 | .286  .452 |
| **RCB Sensory Seek 6-week change**  App Openings  Module Completion | -.008 (.007)  .028 (.016) | -.125  .213 | .292  .083 |
| **RCB Sensory Seek 12-week change**  App Openings  Module Completion | -.017 (.020)  .008 (.011) | -.404  .092 | .7391  .452 |
| **RCB Sensory Avoid 6-week change**  App Openings  Module Completion | .004 (.005)  .001 (.011) | .089  .012 | .464  .922 |
| **RCB Sensory Avoid 12-week change**  App Openings  Module Completion | -.025 (.023)  -.011 (.013) | -.459  -.104 | .286  .366 |
| **PSOC^d^ Efficacy 6-week change**  App Openings  Module Completion | .013 (.004)  -.009 (.009) | .354  -.120 | .002  .301 |
| **PSOC Efficacy 12-week change**  App Openings  Module Completion | .037 (.015)  -.019 (.008) | .794  -.202 | .015  .018 |
| **PSOC Satisfaction 6-week change**  App Openings  Module Completion | -.003 (.005)  -.007 (.012) | -.071  -.074 | .555  .549 |
| **PSOC Satisfaction 12-week change**  App Openings  Module Completion | .021 (.022)  -.009 (.013) | .401  -.085 | .352  .485 |
| **K&A^e^ 12-week change**  App Openings  Module Completion | .026 (.015)  .003 (.007) | .693  .037 | .094  .706 |
| **Secondary Outcomes** |  |  |  |
| **ELS^f^ Responsibility 6-week change**  App Openings  Module Completion | .005 (.008)  .008 (.017) | .069  .059 | .570  .631 |
| **ELS Responsibility 12-week change**  App Openings  Module Completion | .009 (.041)  .014 (.020) | .124  .088 | .822  .506 |
| **ELS Smoothness 6-week change**  App Openings  Module Completion | .007(.009)  .019 (.018) | .093  .128 | .438  .293 |
| **ELS Smoothness 12-week change**  App Openings  Module Completion | .046 (.030)  .020 (.018) | .556  .117 | .126  .260 |

^a^SE = standard error

^b^ECBI = Eyberg Child Behavior Inventory

^c^RCB = Reasons for Child Behavior scale

^d^PSOC = Parenting Sense of Competency Scale

^e^K&A = FASD Knowledge and Advocacy Scale

^f^ELS = Eveyday Life Scale
