# Supplementary material for: Families Moving Forward Connect mHealth Intervention for Caregivers of Children With Fetal Alcohol Spectrum Disorders: Randomized Controlled Trial
Source: JMIR Mhealth Uhealth. 2026 Mar 20;14:e73647. doi: 10.2196/73647 (PMC13004560; doi:10.2196/73647)
Supplement: Checklist 1 [file mhealth-v14-e73647-s004.pdf]

CONSORT-EHEALTH (V 1.6.1) - Submission/Publication Form

The CONSORT-EHEALTH checklist is intended for authors of randomized trials evaluating web-based and Internet-based applications/interventions, including mobile interventions, electronic games (incl multiplayer games), social media, certain telehealth applications, and other interactive and/or networked electronic applications. Some of the items (e.g. all subitems under item 5 - description of the intervention) may also be applicable for other study designs.

The goal of the CONSORT EHEALTH checklist and guideline is to be
a) a guide for reporting for authors of RCTs,
b) to form a basis for appraisal of an ehealth trial (in terms of validity)

CONSORT-EHEALTH items/subitems are MANDATORY reporting items for studies published in the Journal of Medical Internet Research and other journals / scientific societies endorsing the checklist.

Items numbered 1., 2., 3., 4a., 4b etc are original CONSORT or CONSORT-NPT (non-pharmacologic treatment) items.
Items with Roman numerals (i., ii, iii, iv etc.) are CONSORT-EHEALTH extensions/clarifications.

As the CONSORT-EHEALTH checklist is still considered in a formative stage, we would ask that you also RATE ON A SCALE OF 1-5 how important/useful you feel each item is FOR THE PURPOSE OF THE CHECKLIST and reporting guideline (optional).

Mandatory reporting items are marked with a red *.
In the textboxes, either copy & paste the relevant sections from your manuscript into this form - please include any quotes from your manuscript in QUOTATION MARKS,
or answer directly by providing additional information not in the manuscript, or elaborating on why the item was not relevant for this study.

YOUR ANSWERS WILL BE PUBLISHED AS A SUPPLEMENTARY FILE TO YOUR PUBLICATION IN JMIR AND ARE CONSIDERED PART OF YOUR PUBLICATION (IF ACCEPTED).
Please fill in these questions diligently. Information will not be copyedited, so please use proper spelling and grammar, use correct capitalization, and avoid abbreviations.

DO NOT FORGET TO SAVE AS PDF _AND_ CLICK THE SUBMIT BUTTON SO YOUR ANSWERS ARE IN OUR DATABASE !!!

Citation Suggestion (if you append the pdf as Appendix we suggest to cite this paper in the caption):
Eysenbach G, CONSORT-EHEALTH Group

CONSORT-EHEALTH: Improving and Standardizing Evaluation Reports of Web-based and Mobile Health Interventions
J Med Internet Res 2011;13(4):e126
URL: http://www.jmir.org/2011/4/e126/
doi: 10.2196/jmir.1923
PMID: 22209829

sonrisa2134@gmail.com Switch account

Not shared

Draft saved

* Indicates required question

Your name *

First Last

Christie Petrenko

Primary Affiliation (short), City, Country *

University of Toronto, Toronto, Canada

University of Rochester, Rochester, NY USA

Your e-mail address *

abc@gmail.com

christie_petrenko@urmc.rochester.edu

Title of your manuscript *

Provide the (draft) title of your manuscript.

Families Moving Forward Connect Mobile Health Intervention for Caregivers of Children with Fetal Alcohol Spectrum Disorders: Randomized Controlled Trial

Name of your App/Software/Intervention *

If there is a short and a long/alternate name, write the short name first and add the long name in brackets.

FMF Connect [Families Moving Forward Conne

Evaluated Version (if any)

e.g. "V1", "Release 2017-03-01", "Version 2.0.27913"

Your answer

Language(s) *

What language is the intervention/app in? If multiple languages are available, separate by comma (e.g. "English, French")

English

URL of your Intervention Website or App

e.g. a direct link to the mobile app on app in appstore (itunes, Google Play), or URL of the website. If the intervention is a DVD or hardware, you can also link to an Amazon page.

https://www.fmfconnect.com/

URL of an image/screenshot (optional)

Your answer

Accessibility *

Can an enduser access the intervention presently?

- access is free and open
- access only for special usergroups, not open
- access is open to everyone, but requires payment/subscription/in-app purchases
- app/intervention no longer accessible
- (selected) Other: App is currently only accessible for study participants, but will be com

Primary Medical Indication/Disease/Condition *

e.g. "Stress", "Diabetes", or define the target group in brackets after the condition, e.g. "Autism (Parents of children with)", "Alzheimers (Informal Caregivers of)"

fetal alcohol spectrum disorders (Caregivers o

Primary Outcomes measured in trial *

comma-separated list of primary outcomes reported in the trial

child behavior problems, child adaptive functic

Secondary/other outcomes

Are there any other outcomes the intervention is expected to affect?

participant ratings of app quality, patterns of app usage

Recommended "Dose" *

What do the instructions for users say on how often the app should be used?

- ( ) Approximately Daily
- ( ) Approximately Weekly
- ( ) Approximately Monthly
- ( ) Approximately Yearly
- ( ) "as needed"
- (•) Other: user preference

Approx. Percentage of Users (starters) still using the app as recommended after 3 months *

- (•) unknown / not evaluated
- ( ) 0-10%
- ( ) 11-20%
- ( ) 21-30%
- ( ) 31-40%
- ( ) 41-50%
- ( ) 51-60%
- ( ) 61-70%
- ( ) 71%-80%
- ( ) 81-90%
- ( ) 91-100%
- ( ) Other:

Overall, was the app/intervention effective? *

- ( ) yes: all primary outcomes were significantly better in intervention group vs control
- (x) partly: SOME primary outcomes were significantly better in intervention group vs control
- ( ) no statistically significant difference between control and intervention
- ( ) potentially harmful: control was significantly better than intervention in one or more outcomes
- ( ) inconclusive: more research is needed
- ( ) Other:

Article Preparation Status/Stage *

At which stage in your article preparation are you currently (at the time you fill in this form)

- ( ) not submitted yet - in early draft status
- (x) not submitted yet - in late draft status, just before submission
- ( ) submitted to a journal but not reviewed yet
- ( ) submitted to a journal and after receiving initial reviewer comments
- ( ) submitted to a journal and accepted, but not published yet
- ( ) published
- ( ) Other:

Journal *

If you already know where you will submit this paper (or if it is already submitted), please provide the journal name (if it is not JMIR, provide the journal name under "other")

- ○ not submitted yet / unclear where I will submit this
- ○ Journal of Medical Internet Research (JMIR)
- ◉ JMIR mHealth and UHealth
- ○ JMIR Serious Games
- ○ JMIR Mental Health
- ○ JMIR Public Health
- ○ JMIR Formative Research
- ○ Other JMIR sister journal
- ○ Other:

Is this a full powered effectiveness trial or a pilot/feasibility trial? *

- ○ Pilot/feasibility
- ◉ Fully powered

Manuscript tracking number *

If this is a JMIR submission, please provide the manuscript tracking number under "other" (The ms tracking number can be found in the submission acknowledgement email, or when you login as author in JMIR. If the paper is already published in JMIR, then the ms tracking number is the four-digit number at the end of the DOI, to be found at the bottom of each published article in JMIR)

- ◉ no ms number (yet) / not (yet) submitted to / published in JMIR
- ○ Other:

TITLE AND ABSTRACT

1a) TITLE: Identification as a randomized trial in the title

1a) Does your paper address CONSORT item 1a? *

I.e does the title contain the phrase "Randomized Controlled Trial"? (if not, explain the reason under "other")

- (•) yes
- ( ) Other:

1a-i) Identify the mode of delivery in the title

Identify the mode of delivery. Preferably use “web-based” and/or “mobile” and/or “electronic game” in the title. Avoid ambiguous terms like “online”, “virtual”, “interactive”. Use “Internet-based” only if Intervention includes non-web-based Internet components (e.g. email), use “computer-based” or “electronic” only if offline products are used. Use “virtual” only in the context of “virtual reality” (3-D worlds). Use “online” only in the context of “online support groups”. Complement or substitute product names with broader terms for the class of products (such as “mobile” or “smart phone” instead of “iphone”), especially if the application runs on different platforms.

	1	2	3	4	5	
subitem not at all important	( )	( )	( )	( )	(•)	essential

Clear selection

Does your paper address subitem 1a-i? *

Copy and paste relevant sections from manuscript title (include quotes in quotation marks "like this" to indicate direct quotes from your manuscript), or elaborate on this item by providing additional information not in the ms, or briefly explain why the item is not applicable/relevant for your study

"Families Moving Forward Connect Mobile Health Intervention for Caregivers of Children with Fetal Alcohol Spectrum Disorders: Randomized Controlled Trial"

1a-ii) Non-web-based components or important co-interventions in title

Mention non-web-based components or important co-interventions in title, if any (e.g., “with telephone support”).

	1	2	3	4	5	
subitem not at all important	○	○	○	●	○	essential

Clear selection

Does your paper address subitem 1a-ii?

Copy and paste relevant sections from manuscript title (include quotes in quotation marks "like this" to indicate direct quotes from your manuscript), or elaborate on this item by providing additional information not in the ms, or briefly explain why the item is not applicable/relevant for your study

FMF Connect does not include any non-web-based components or co-interventions

1a-iii) Primary condition or target group in the title

Mention primary condition or target group in the title, if any (e.g., “for children with Type I Diabetes”) Example: A Web-based and Mobile Intervention with Telephone Support for Children with Type I Diabetes: Randomized Controlled Trial

	1	2	3	4	5	
subitem not at all important	○	○	○	○	◉	essential

Clear selection

Does your paper address subitem 1a-iii? *

Copy and paste relevant sections from manuscript title (include quotes in quotation marks "like this" to indicate direct quotes from your manuscript), or elaborate on this item by providing additional information not in the ms, or briefly explain why the item is not applicable/relevant for your study

"Families Moving Forward Connect Mobile Health Intervention for Caregivers of Children with Fetal Alcohol Spectrum Disorders: Randomized Controlled Trial"

1b) ABSTRACT: Structured summary of trial design, methods, results, and conclusions

NPT extension: Description of experimental treatment, comparator, care providers, centers, and blinding status.

1b-i) Key features/functionalities/components of the intervention and comparator in the METHODS section of the ABSTRACT

Mention key features/functionalities/components of the intervention and comparator in the abstract. If possible, also mention theories and principles used for designing the site. Keep in mind the needs of systematic reviewers and indexers by including important synonyms. (Note: Only report in the abstract what the main paper is reporting. If this information is missing from the main body of text, consider adding it)

	1	2	3	4	5	
subitem not at all important	○	○	○	◉	○	essential

Clear selection

Does your paper address subitem 1b-i? *

Copy and paste relevant sections from the manuscript abstract (include quotes in quotation marks "like this" to indicate direct quotes from your manuscript), or elaborate on this item by providing additional information not in the ms, or briefly explain why the item is not applicable/relevant for your study

Background: "The Families Moving Forward (FMF) Connect app is a self-directed intervention derived from an empirically-supported, specialist-led intervention for caregivers raising children with FASD. "
Methods: "This study involved a 3-arm randomized controlled trial with equal allocation to groups: 1) FMF Connect + text-based coaching, 2) FMF Connect, or 3) waitlist control group."

1b-ii) Level of human involvement in the METHODS section of the ABSTRACT

Clarify the level of human involvement in the abstract, e.g., use phrases like “fully automated” vs. “therapist/nurse/care provider/physician-assisted” (mention number and expertise of providers involved, if any). (Note: Only report in the abstract what the main paper is reporting. If this information is missing from the main body of text, consider adding it)

	1	2	3	4	5	
subitem not at all important	○	○	○	◉	○	essential

Clear selection

Does your paper address subitem 1b-ii?

Copy and paste relevant sections from the manuscript abstract (include quotes in quotation marks "like this" to indicate direct quotes from your manuscript), or elaborate on this item by providing additional information not in the ms, or briefly explain why the item is not applicable/relevant for your study

Background: "The Families Moving Forward (FMF) Connect app is a self-directed intervention derived from an empirically-supported, specialist-led intervention for caregivers raising children with FASD. "
Methods: "This study involved a 3-arm randomized controlled trial with equal allocation to groups: 1) FMF Connect + text-based coaching, 2) FMF Connect, or 3) waitlist control group."

1b-iii) Open vs. closed, web-based (self-assessment) vs. face-to-face assessments in the METHODS section of the ABSTRACT

Mention how participants were recruited (online vs. offline), e.g., from an open access website or from a clinic or a closed online user group (closed usergroup trial), and clarify if this was a purely web-based trial, or there were face-to-face components (as part of the intervention or for assessment). Clearly say if outcomes were self-assessed through questionnaires (as common in web-based trials). Note: In traditional offline trials, an open trial (open-label trial) is a type of clinical trial in which both the researchers and participants know which treatment is being administered. To avoid confusion, use “blinded” or “unblinded” to indicated the level of blinding instead of “open”, as “open” in web-based trials usually refers to “open access” (i.e. participants can self-enrol). (Note: Only report in the abstract what the main paper is reporting. If this information is missing from the main body of text, consider adding it)

	1	2	3	4	5	
subitem not at all important	○	○	○	◉	○	essential

Clear selection

Does your paper address subitem 1b-iii?

Copy and paste relevant sections from the manuscript abstract (include quotes in quotation marks "like this" to indicate direct quotes from your manuscript), or elaborate on this item by providing additional information not in the ms, or briefly explain why the item is not applicable/relevant for your study

"Participants were recruited online through an open access website. Recruitment materials were distributed by investigators in the Collaborative Initiative on FASD (CIFASD), FASD listservs, and social media."

1b-iv) RESULTS section in abstract must contain use data

Report number of participants enrolled/assessed in each group, the use/uptake of the intervention (e.g., attrition/adherence metrics, use over time, number of logins etc.), in addition to primary/secondary outcomes. (Note: Only report in the abstract what the main paper is reporting. If this information is missing from the main body of text, consider adding it)

	1	2	3	4	5	
subitem not at all important	○	○	◉	○	○	essential

Clear selection

Does your paper address subitem 1b-iv?

Copy and paste relevant sections from the manuscript abstract (include quotes in quotation marks "like this" to indicate direct quotes from your manuscript), or elaborate on this item by providing additional information not in the ms, or briefly explain why the item is not applicable/relevant for your study

"Results: 43 participants were randomized to each group. 64 percent of participants were retained through the 12-week follow-up. Groups did not generally differ in terms of demographic characteristics, baseline levels of functioning, or patterns of attrition. Usage patterns were similar across intervention groups, suggesting coaching did not increase app engagement."

1b-v) CONCLUSIONS/DISCUSSION in abstract for negative trials

Conclusions/Discussions in abstract for negative trials: Discuss the primary outcome - if the trial is negative (primary outcome not changed), and the intervention was not used, discuss whether negative results are attributable to lack of uptake and discuss reasons. (Note: Only report in the abstract what the main paper is reporting. If this information is missing from the main body of text, consider adding it)

	1	2	3	4	5	
subitem not at all important	○	○	○	◉	○	essential

Clear selection

Does your paper address subitem 1b-v?

Copy and paste relevant sections from the manuscript abstract (include quotes in quotation marks "like this" to indicate direct quotes from your manuscript), or elaborate on this item by providing additional information not in the ms, or briefly explain why the item is not applicable/relevant for your study

"Relative to the waitlist comparison group, caregivers who received the FMF Connect app evidenced greater improvements in child behavior attributions, parenting satisfaction, and family needs met after 12 weeks ($p = .011 - .044$). After controlling for multiple comparisons, differences in family needs met approached significance ($p = 0.06$). Groups did not differ in parenting efficacy, self-care, or child behavior problems or adaptive functioning. Caregiver behavior attributions at 6-weeks did not significantly mediate intervention effects."

INTRODUCTION

2a) In INTRODUCTION: Scientific background and explanation of rationale

2a-i) Problem and the type of system/solution

Describe the problem and the type of system/solution that is object of the study: intended as stand-alone intervention vs. incorporated in broader health care program? Intended for a particular patient population? Goals of the intervention, e.g., being more cost-effective to other interventions, replace or complement other solutions? (Note: Details about the intervention are provided in “Methods” under 5)

	1	2	3	4	5	
subitem not at all important	○	○	○	○	◉	essential

Clear selection

Does your paper address subitem 2a-i? *

Copy and paste relevant sections from the manuscript (include quotes in quotation marks "like this" to indicate direct quotes from your manuscript), or elaborate on this item by providing additional information not in the ms, or briefly explain why the item is not applicable/relevant for your study

"Despite higher needs, individuals with FASD and their families report substantial barriers to services due to limited availability of knowledgeable providers, societal stigma, lack of policy-based supports, and insufficient availability of appropriate, evidence-based services (McCormack 2021). These systematic barriers emphasize the need for evidence-based interventions that are both accessible and tailored to the needs of families managing FASD, presenting an opportunity to apply innovative methods and new implementation strategies. One method to surmount these barriers is mobile delivery of services or app-based interventions."

"As mHealth apps offer a promising solution to many barriers faced by families and providers when trying to access FASD-informed care, the FMF Connect app was developed to provide accessible, evidence-based support for caregivers of children with FASD. The FMF Connect app is the first known stand-alone, self-directed parenting app to be empirically evaluated for efficacy for caregiver and child outcomes."

2a-ii) Scientific background, rationale: What is known about the (type of) system

Scientific background, rationale: What is known about the (type of) system that is the object of the study (be sure to discuss the use of similar systems for other conditions/diagnoses, if appropiate), motivation for the study, i.e. what are the reasons for and what is the context for this specific study, from which stakeholder viewpoint is the study performed, potential impact of findings [2]. Briefly justify the choice of the comparator.

	1	2	3	4	5	
subitem not at all important	○	○	○	○	○	essential

Does your paper address subitem 2a-ii? *

Copy and paste relevant sections from the manuscript (include quotes in quotation marks "like this" to indicate direct quotes from your manuscript), or elaborate on this item by providing additional information not in the ms, or briefly explain why the item is not applicable/relevant for your study

"Despite the promise of app-based interventions, there have been very few mHealth apps with evidence-based review that follows recommended mental health app guidelines (Bakker et al., 2016). Yet the market is inundated with apps that vary highly in quality, user engagement tools, and evidence-based content, leading to cycles of trial and error in order to find one that fits user needs (Shorey et al. 2023; Virani et al. 2019) and potentially straining already limited caregiver resources. A meta-review of parenting-based technological interventions by David and colleagues (2024) indicated that of the 53 usable parenting-focused apps available in app stores, few offered personalized content or focused on caregiver functioning, and none have yet conducted a randomized controlled trial (RCT)."

2b) In INTRODUCTION: Specific objectives or hypotheses

Does your paper address CONSORT subitem 2b? *

Copy and paste relevant sections from the manuscript (include quotes in quotation marks "like this" to indicate direct quotes from your manuscript), or elaborate on this item by providing additional information not in the ms, or briefly explain why the item is not applicable/relevant for your study

"The primary objective of the current study was to determine if the FMF Connect app was related to improved targeted caregiver and child outcomes over a 12-week period. This study involved a 3-arm parallel RCT with equal allocation to three study groups who received: 1) FMF Connect app plus text-based coaching to support app engagement; 2) FMF Connect app without coaching; and 3) waitlist control group who received the app after study conclusion. Relative to the waitlist control group, we hypothesized that assignment to the FMF Connect groups would be associated with increases in caregiver FASD knowledge, improvements in parenting efficacy and satisfaction, reductions in caregiver attributions of willful child behavior, increases in family needs met, and improved child behavior. We hypothesized that the addition of text-based coaching would result in greater app usage, and, in turn, greater change across outcomes. A 6-week timepoint was also included to test whether improvements in caregiver attributions of child behavior would mediate intervention effects on child behavior and caregiver self-efficacy and satisfaction. We also assessed caregiver perceptions of app quality, consistent with prior feasibility trials (Petrenko et al., 2024)."

METHODS

3a) Description of trial design (such as parallel, factorial) including allocation ratio

Does your paper address CONSORT subitem 3a? *

Copy and paste relevant sections from the manuscript (include quotes in quotation marks "like this" to indicate direct quotes from your manuscript), or elaborate on this item by providing additional information not in the ms, or briefly explain why the item is not applicable/relevant for your study

"This study involved a three parallel arm randomized controlled trial with equal allocation to the following conditions: (1) FMF Connect + Coaching; (2) FMF Connect without coaching; and (3) waitlist control. Quantitative survey data was collected at three timepoints: baseline, 6-weeks, and 12-weeks. The 6-week timepoint involved an abbreviated battery designed to allow for tests of statistical mediation hypotheses. App usage was recorded through Amazon Web Services (AWS) during the study period. After 12 weeks, study participants in the waitlist control group received the FMF Connect app."

3b) Important changes to methods after trial commencement (such as eligibility criteria), with reasons

Does your paper address CONSORT subitem 3b? *

Copy and paste relevant sections from the manuscript (include quotes in quotation marks "like this" to indicate direct quotes from your manuscript), or elaborate on this item by providing additional information not in the ms, or briefly explain why the item is not applicable/relevant for your study

"No changes were made to the trial after initiation."

3b-i) Bug fixes, Downtimes, Content Changes

Bug fixes, Downtimes, Content Changes: ehealth systems are often dynamic systems. A description of changes to methods therefore also includes important changes made on the intervention or comparator during the trial (e.g., major bug fixes or changes in the functionality or content) (5-iii) and other “unexpected events” that may have influenced study design such as staff changes, system failures/downtimes, etc. [2].

	1	2	3	4	5	
subitem not at all important	○	○	◉	○	○	essential

Clear selection

Does your paper address subitem 3b-i?

Copy and paste relevant sections from the manuscript (include quotes in quotation marks "like this" to indicate direct quotes from your manuscript), or elaborate on this item by providing additional information not in the ms, or briefly explain why the item is not applicable/relevant for your study

"Four minor updates were made to the app during the first two months of the trial. These included small wording changes, optimization of the connection with the backend system, adding banners to the Family Forum to easily identify moderators, fixed an issue with the behavior tracker tool affecting some users, added new notification management system, and minor bug fixes for performance improvements."

4a) Eligibility criteria for participants

Does your paper address CONSORT subitem 4a? *

Copy and paste relevant sections from the manuscript (include quotes in quotation marks "like this" to indicate direct quotes from your manuscript), or elaborate on this item by providing additional information not in the ms, or briefly explain why the item is not applicable/relevant for your study

"Inclusion criteria:
Biological parent or other primary caregiver (e.g., foster or adoptive parent, relative, legal guardian) who:
• Is at least 18 years old
• Lives in the United States
• Has a smartphone or iPad with iOS operating system
• Has a child who:
o Has a diagnosis of FASD or confirmed PAE. As part of study registration, caregivers completed a survey about the child's PAE and uploaded diagnostic records, if available. The study team reviewed available information and determined study eligibility.
o Is between the ages of 3 and 12 years old. If there was more than one child in the family with FASD/PAE in this age range, the caregiver was instructed during screening to choose one child as the target of study surveys.
o Has lived with the caregiver for at least 4 months and is expected to remain in the home for at least 1 year

Exclusion criteria:
• The caregiver is not fluent in English (the FMF Connect app and measures are currently only available in English)
• There is another caregiver of the same child or living in the home who is already enrolled in the study (couples are excluded to prevent dependence in the data)
• The caregiver participated in a prior trial of the FMF Connect app as part of earlier development phases of this project (Petrenko et al., 2020, 2021, 2024).
• The family has previously received or is currently receiving the specialist-led standard FMF Program "

4a-i) Computer / Internet literacy

Computer / Internet literacy is often an implicit "de facto" eligibility criterion - this should be explicitly clarified.

	1	2	3	4	5	
subitem not at all important	○	○	◉	○	○	essential

Clear selection

Does your paper address subitem 4a-i?

Copy and paste relevant sections from the manuscript (include quotes in quotation marks "like this" to indicate direct quotes from your manuscript), or elaborate on this item by providing additional information not in the ms, or briefly explain why the item is not applicable/relevant for your study

Participants needed to own a smartphone or ipad with iOS operating system to be eligible. Basic usage of these devices was assumed.

4a-ii) Open vs. closed, web-based vs. face-to-face assessments:

Open vs. closed, web-based vs. face-to-face assessments: Mention how participants were recruited (online vs. offline), e.g., from an open access website or from a clinic, and clarify if this was a purely web-based trial, or there were face-to-face components (as part of the intervention or for assessment), i.e., to what degree got the study team to know the participant. In online-only trials, clarify if participants were quasi-anonymous and whether having multiple identities was possible or whether technical or logistical measures (e.g., cookies, email confirmation, phone calls) were used to detect/prevent these.

	1	2	3	4	5	
subitem not at all important	○	○	○	○	◉	essential

Clear selection

Does your paper address subitem 4a-ii? *

Copy and paste relevant sections from the manuscript (include quotes in quotation marks "like this" to indicate direct quotes from your manuscript), or elaborate on this item by providing additional information not in the ms, or briefly explain why the item is not applicable/relevant for your study

"Recruitment, informed consent, and data collection procedures were conducted online."
"Participants were recruited through a variety of mechanisms by our study team and collaborators within the Collaborative Initiative on FASD (CIFASD; www.cifasd.org) and community partners. These included disseminating recruitment materials on social media, through national and regional FASD listservs (e.g., FASD United's Weekly Round-up), at relevant conferences, and to participants or patients with whom study team or collaborators had routine access and/or had agreed to contact about new research opportunities.

Study recruitment materials included links to the study website (www.fmfconnect.com), where potential participants could access study information and link to the study's Consenting and Screening module. All surveys were administered via REDCap, hosted by the University of Rochester, and REDCap's eConsent package was used to document participant's informed consent. Participants had the option to review study consent information by video or text and were required to answer several questions to ensure comprehension. Participants were also offered the opportunity to ask questions via direct contact with the study coordinator before proceeding to other parts of the survey.

After completing informed consent, participants were directed in REDCap to answer screening questions to assess for study eligibility, including information about PAE, FASD diagnostic records, and demographic information. The study team determined eligibility and assigned a globally unique identifier (GUID) to each eligible caregiver participant and child to facilitate data sharing within CIFASD or future studies. Eligible participants then received an email inviting them to complete baseline study surveys."

4a-iii) Information giving during recruitment

Information given during recruitment. Specify how participants were briefed for recruitment and in the informed consent procedures (e.g., publish the informed consent documentation as appendix, see also item X26), as this information may have an effect on user self-selection, user expectation and may also bias results.

	1	2	3	4	5	
subitem not at all important	○	○	○	○	◉	essential

Clear selection

Does your paper address subitem 4a-iii?

Copy and paste relevant sections from the manuscript (include quotes in quotation marks "like this" to indicate direct quotes from your manuscript), or elaborate on this item by providing additional information not in the ms, or briefly explain why the item is not applicable/relevant for your study

"Study recruitment materials included links to the study website (www.fmfconnect.com), where potential participants could access study information and link to the study's Consenting and Screening module. All surveys were administered via REDCap, hosted by the University of Rochester, and REDCap's eConsent package was used to document participant's informed consent. Participants had the option to review study consent information by video or text and were required to answer several questions to ensure comprehension. Participants were also offered the opportunity to ask questions via direct contact with the study coordinator before proceeding to other parts of the survey."

4b) Settings and locations where the data were collected

Does your paper address CONSORT subitem 4b? *

Copy and paste relevant sections from the manuscript (include quotes in quotation marks "like this" to indicate direct quotes from your manuscript), or elaborate on this item by providing additional information not in the ms, or briefly explain why the item is not applicable/relevant for your study

"Data collection procedures were conducted online."
"All surveys were administered via REDCap, hosted by the University of Rochester"

4b-i) Report if outcomes were (self-)assessed through online questionnaires

Clearly report if outcomes were (self-)assessed through online questionnaires (as common in web-based trials) or otherwise.

	1	2	3	4	5	
subitem not at all important	○	○	○	◉	○	essential

Clear selection

Does your paper address subitem 4b-i? *

Copy and paste relevant sections from the manuscript (include quotes in quotation marks "like this" to indicate direct quotes from your manuscript), or elaborate on this item by providing additional information not in the ms, or briefly explain why the item is not applicable/relevant for your study

Primary outcomes were all assessed through self-report questionnaires completed by participants. Usage data was also collected through AWS.

4b-ii) Report how institutional affiliations are displayed

Report how institutional affiliations are displayed to potential participants [on ehealth media], as affiliations with prestigious hospitals or universities may affect volunteer rates, use, and reactions with regards to an intervention.(Not a required item – describe only if this may bias results)

	1	2	3	4	5	
subitem not at all important	○	○	○	○	○	essential

Does your paper address subitem 4b-ii?

Copy and paste relevant sections from the manuscript (include quotes in quotation marks "like this" to indicate direct quotes from your manuscript), or elaborate on this item by providing additional information not in the ms, or briefly explain why the item is not applicable/relevant for your study

Your answer

5) The interventions for each group with sufficient details to allow replication, including how and when they were actually administered

5-i) Mention names, credential, affiliations of the developers, sponsors, and owners

Mention names, credential, affiliations of the developers, sponsors, and owners [6] (if authors/evaluators are owners or developer of the software, this needs to be declared in a “Conflict of interest” section or mentioned elsewhere in the manuscript).

	1	2	3	4	5	
subitem not at all important	○	○	○	◉	○	essential

Clear selection

Does your paper address subitem 5-i?

Copy and paste relevant sections from the manuscript (include quotes in quotation marks "like this" to indicate direct quotes from your manuscript), or elaborate on this item by providing additional information not in the ms, or briefly explain why the item is not applicable/relevant for your study

"FMF Connect Intervention
FMF Connect includes cloud infrastructure and an innovative, multilayered mHealth app. It incorporates tailored content for caregivers of children (ages 3-12) with FASD or PAE. The app integrates five main components: 1) Dashboard; 2) Learning Modules; 3) Family Forum; 4) Library; and 5) Notebook (see Figure 1). Weekly emails were also sent to support motivational engagement.

App education, derived from the standard FMF Program, is packaged in easily digestible Learning Modules. The Learning Modules are organized into three levels: 1) Getting Started; 2) Taking Action; and 3) Taking It to the Next Level. The Library provides access to additional optional content that can be downloaded and printed to use in advocacy and skill development. The Notebook is a personalized section that organizes caregivers' responses to learning activities in a convenient location for later easy access.

The Family Forum serves to: 1) engage and facilitate caregivers' continued utilization of the app; 2) support and extend caregivers' implementation of new knowledge and skills; and 3) provide peer support, encouragement, and understanding of the caregiver's own needs. Trained peer moderators moderated discussion in family forums to ensure safety. They also provided support and prompt skills or content areas to consider.

The Dashboard displays caregiver progress in Learning Module completion, use of the Family Forum, and daily and weekly ratings imputed by the caregiver. It also includes the Tip of the Day feature.

Complementing the app is a Cloud infrastructure to transparently but securely distribute information, including storing and retrieving data, managing notification and messaging, and synchronizing data on all devices. A local datastore protects the mobile app against network failures and connectivity issues. This local datastore is transparently synchronized with the Cloud database so that all querying and security features are constantly available regardless of network connectivity. Updates are automatically pushed so users have the latest version.

Coaching Module
A Coaching Module was developed within the FMF Connect app to help engage caregivers and support continued use and individualized goal setting within the app. The Coaching Module was activated for caregivers in the FMF Connect + Coaching group only. This Module allowed an assigned coach to exchange text-based messages with caregivers through the FMF Connect app's notification system. Coaches had access to a dashboard of their participants' progress and use of the app, which aided in tailoring messages and supporting caregivers in their identified goals. The coach's interface also provided a secure way to provide text-based messages to the participant within the app.

Coaches were graduate students on the project familiar with FMF Program principles and knowledgeable about FMF Connect content and features. They had training in motivational interviewing (MI), which is a key therapeutic technique used in the FMF Program. Coaches used the spirit and skills of MI to evoke the participant's own motivation to learn more about FASD and skills taught in FMF Connect. They also aimed to support caregiver's goals by helping link them with content and features within FMF Connect."

5-ii) Describe the history/development process

Describe the history/development process of the application and previous formative evaluations (e.g., focus groups, usability testing), as these will have an impact on adoption/use rates and help with interpreting results.

	1	2	3	4	5	
subitem not at all important	○	○	○	◉	○	essential

Clear selection

Does your paper address subitem 5-ii?

Copy and paste relevant sections from the manuscript (include quotes in quotation marks "like this" to indicate direct quotes from your manuscript), or elaborate on this item by providing additional information not in the ms, or briefly explain why the item is not applicable/relevant for your study

"The FMF Connect app is the first known stand-alone, self-directed parenting app to be empirically evaluated for efficacy for caregiver and child outcomes (Petrenko et al., 2020, 2021, 2024). The app was derived from the Families Moving Forward (FMF) Program, a 14- to 17-session program for caregivers of children aged 3-12 years with FASD and challenging behavior problems delivered by a trained specialist (Bertrand 2009). This program was developed and tested by Dr. Heather Carmichael Olson and colleagues at Seattle Children's Research Institute and the University of Washington, with funding from the Centers for Disease Control."
"The systematic user-centered design approach to develop and test the FMF Connect app has been relatively rare in the field of mHealth apps. This approach, detailed in Petrenko et al. (2021), emphasizes iterative feedback at each stage of development from community members who represent target users for the app. This process has included focus groups with caregivers on the initial design and functionalities of the proposed app (Petrenko et al., 2020), interviews with caregivers and providers following beta-testing of initial app prototypes (Petrenko et al., 2021), and surveys and interviews with caregivers during a larger-scale feasibility trial (Petrenko et al., 2024). The current study represents the next critical phase of our systematic evaluation, which is to test the efficacy of the app in an RCT."

5-iii) Revisions and updating

Revisions and updating. Clearly mention the date and/or version number of the application/intervention (and comparator, if applicable) evaluated, or describe whether the intervention underwent major changes during the evaluation process, or whether the development and/or content was “frozen” during the trial. Describe dynamic components such as news feeds or changing content which may have an impact on the replicability of the intervention (for unexpected events see item 3b).

	1	2	3	4	5	
subitem not at all important	○	○	○	○	○	essential

Does your paper address subitem 5-iii?

Copy and paste relevant sections from the manuscript (include quotes in quotation marks "like this" to indicate direct quotes from your manuscript), or elaborate on this item by providing additional information not in the ms, or briefly explain why the item is not applicable/relevant for your study

"The systematic user-centered design approach to develop and test the FMF Connect app has been relatively rare in the field of mHealth apps. This approach, detailed in Petrenko et al. (2021), emphasizes iterative feedback at each stage of development from community members who represent target users for the app. This process has included focus groups with caregivers on the initial design and functionalities of the proposed app (Petrenko et al., 2020), interviews with caregivers and providers following beta-testing of initial app prototypes (Petrenko et al., 2021), and surveys and interviews with caregivers during a larger-scale feasibility trial (Petrenko et al., 2024)."
No major changes were made to the app during the RCT.

5-iv) Quality assurance methods

Provide information on quality assurance methods to ensure accuracy and quality of information provided [1], if applicable.

	1	2	3	4	5	
subitem not at all important	○	○	○	○	○	essential

Does your paper address subitem 5-iv?

Copy and paste relevant sections from the manuscript (include quotes in quotation marks "like this" to indicate direct quotes from your manuscript), or elaborate on this item by providing additional information not in the ms, or briefly explain why the item is not applicable/relevant for your study

It is not entirely clear what this item is requesting.

5-v) Ensure replicability by publishing the source code, and/or providing screenshots/screen-capture video, and/or providing flowcharts of the algorithms used

Ensure replicability by publishing the source code, and/or providing screenshots/screen-capture video, and/or providing flowcharts of the algorithms used. Replicability (i.e., other researchers should in principle be able to replicate the study) is a hallmark of scientific reporting.

	1	2	3	4	5	
subitem not at all important	○	○	●	○	○	essential

Clear selection

Does your paper address subitem 5-v?

Copy and paste relevant sections from the manuscript (include quotes in quotation marks "like this" to indicate direct quotes from your manuscript), or elaborate on this item by providing additional information not in the ms, or briefly explain why the item is not applicable/relevant for your study

A screenshot of the learning modules and description of other app features are included in Figure 1.
Our website and prior manuscripts developing the app include additional screenshots.

5-vi) Digital preservation

Digital preservation: Provide the URL of the application, but as the intervention is likely to change or disappear over the course of the years; also make sure the intervention is archived (Internet Archive, webcitation.org, and/or publishing the source code or screenshots/videos alongside the article). As pages behind login screens cannot be archived, consider creating demo pages which are accessible without login.

	1	2	3	4	5	
subitem not at all important	○	○	○	○	○	essential

Does your paper address subitem 5-vi?

Copy and paste relevant sections from the manuscript (include quotes in quotation marks "like this" to indicate direct quotes from your manuscript), or elaborate on this item by providing additional information not in the ms, or briefly explain why the item is not applicable/relevant for your study

The URL of the application is: www.fmfconnect.com

5-vii) Access

Access: Describe how participants accessed the application, in what setting/context, if they had to pay (or were paid) or not, whether they had to be a member of specific group. If known, describe how participants obtained "access to the platform and Internet" [1]. To ensure access for editors/reviewers/readers, consider to provide a "backdoor" login account or demo mode for reviewers/readers to explore the application (also important for archiving purposes, see vi).

	1	2	3	4	5	
subitem not at all important	○	○	○	○	○	essential

Does your paper address subitem 5-vii? *

Copy and paste relevant sections from the manuscript (include quotes in quotation marks "like this" to indicate direct quotes from your manuscript), or elaborate on this item by providing additional information not in the ms, or briefly explain why the item is not applicable/relevant for your study

"Participants in the intervention groups were given a username and password and were given instructions on how to install the app from the Apple app store."

5-viii) Mode of delivery, features/functionalities/components of the intervention and comparator, and the theoretical framework

Describe mode of delivery, features/functionalities/components of the intervention and comparator, and the theoretical framework [6] used to design them (instructional strategy [1], behaviour change techniques, persuasive features, etc., see e.g., [7, 8] for terminology). This includes an in-depth description of the content (including where it is coming from and who developed it) [1],” whether [and how] it is tailored to individual circumstances and allows users to track their progress and receive feedback” [6]. This also includes a description of communication delivery channels and – if computer-mediated communication is a component – whether communication was synchronous or asynchronous [6]. It also includes information on presentation strategies [1], including page design principles, average amount of text on pages, presence of hyperlinks to other resources, etc. [1].

	1	2	3	4	5	
subitem not at all important	○	○	○	○	○	essential

Does your paper address subitem 5-viii? *

Copy and paste relevant sections from the manuscript (include quotes in quotation marks "like this" to indicate direct quotes from your manuscript), or elaborate on this item by providing additional information not in the ms, or briefly explain why the item is not applicable/relevant for your study

"There were no costs to participants for participating in the study. They received the following payments for completing surveys: $40 at baseline, $20 at 6 weeks, and $40 at 12 weeks."

5-ix) Describe use parameters

Describe use parameters (e.g., intended “doses” and optimal timing for use). Clarify what instructions or recommendations were given to the user, e.g., regarding timing, frequency, heaviness of use, if any, or was the intervention used ad libitum.

	1	2	3	4	5	
subitem not at all important	○	○	○	○	○	essential

Does your paper address subitem 5-ix?

Copy and paste relevant sections from the manuscript (include quotes in quotation marks "like this" to indicate direct quotes from your manuscript), or elaborate on this item by providing additional information not in the ms, or briefly explain why the item is not applicable/relevant for your study

Not applicable. Caregivers were free to use the app how they chose.

5-x) Clarify the level of human involvement

Clarify the level of human involvement (care providers or health professionals, also technical assistance) in the e-intervention or as co-intervention (detail number and expertise of professionals involved, if any, as well as “type of assistance offered, the timing and frequency of the support, how it is initiated, and the medium by which the assistance is delivered”. It may be necessary to distinguish between the level of human involvement required for the trial, and the level of human involvement required for a routine application outside of a RCT setting (discuss under item 21 – generalizability).

	1	2	3	4	5	
subitem not at all important	○	○	○	○	○	essential

Does your paper address subitem 5-x?

Copy and paste relevant sections from the manuscript (include quotes in quotation marks "like this" to indicate direct quotes from your manuscript), or elaborate on this item by providing additional information not in the ms, or briefly explain why the item is not applicable/relevant for your study

All caregivers in the intervention groups had access to the Family Forum which allowed caregivers to connect with one another. Trained peer moderators participated in the Forum.

The FMF Connect + Coaching group also were assigned a coach:
"The Coaching Module was activated for caregivers in the FMF Connect + Coaching group only. This Module allowed an assigned coach to exchange text-based messages with caregivers through the FMF Connect app's notification system. Coaches had access to a dashboard of their participants' progress and use of the app, which aided in tailoring messages and supporting caregivers in their identified goals. The coach's interface also provided a secure way to provide text-based messages to the participant within the app.

Coaches were graduate students on the project familiar with FMF Program principles and knowledgeable about FMF Connect content and features. They had training in motivational interviewing (MI), which is a key therapeutic technique used in the FMF Program. Coaches used the spirit and skills of MI to evoke the participant's own motivation to learn more about FASD and skills taught in FMF Connect. They also aimed to support caregiver's goals by helping link them with content and features within FMF Connect."

5-xi) Report any prompts/reminders used

Report any prompts/reminders used: Clarify if there were prompts (letters, emails, phone calls, SMS) to use the application, what triggered them, frequency etc. It may be necessary to distinguish between the level of prompts/reminders required for the trial, and the level of prompts/reminders for a routine application outside of a RCT setting (discuss under item 21 – generalizability).

	1	2	3	4	5	
subitem not at all important	○	○	○	○	○	essential

Does your paper address subitem 5-xi? *

Copy and paste relevant sections from the manuscript (include quotes in quotation marks "like this" to indicate direct quotes from your manuscript), or elaborate on this item by providing additional information not in the ms, or briefly explain why the item is not applicable/relevant for your study

For both intervention groups:
"Weekly emails were also sent to support motivational engagement."
Participants also received a "Tip of the Day" in their Dashboard and via notification.
Participants in the Coaching group also received notifications within the app from their coaches.

5-xii) Describe any co-interventions (incl. training/support)

Describe any co-interventions (incl. training/support): Clearly state any interventions that are provided in addition to the targeted eHealth intervention, as ehealth intervention may not be designed as stand-alone intervention. This includes training sessions and support [1]. It may be necessary to distinguish between the level of training required for the trial, and the level of training for a routine application outside of a RCT setting (discuss under item 21 – generalizability.

	1	2	3	4	5	
subitem not at all important	○	○	○	○	○	essential

Does your paper address subitem 5-xii? *

Copy and paste relevant sections from the manuscript (include quotes in quotation marks "like this" to indicate direct quotes from your manuscript), or elaborate on this item by providing additional information not in the ms, or briefly explain why the item is not applicable/relevant for your study

This item is not applicable

6a) Completely defined pre-specified primary and secondary outcome measures, including how and when they were assessed

Does your paper address CONSORT subitem 6a? *

Copy and paste relevant sections from the manuscript (include quotes in quotation marks "like this" to indicate direct quotes from your manuscript), or elaborate on this item by providing additional information not in the ms, or briefly explain why the item is not applicable/relevant for your study

"Outcomes
Table 1 lists all study surveys administered per timepoint. Primary study outcomes included child behavior problems (ECBI), child adaptive functioning (ELS), caregiver FASD knowledge (K&A), parenting self-efficacy and satisfaction (PSOC), attributions of child behavior, family needs met (FNM), and caregiver use of self-care. We also examined participant ratings of app quality (uMARS) and patterns of app usage (from AWS). Additional measures of family background and services were included to characterize the sample. T

Table 1. Study Assessment Battery
Measure Baseline 6-
Weeks 12-Weeks
Eyberg Child Behavior Inventory (ECBI) X X
Reasons for Children's Behavior Scale (RCB) X X X
Everyday Life Scale (ELS) X X X
Parenting Sense of Competence Scale (PSOC) X X X
Family Needs Questionnaire (FNM) X X
Knowledge & Advocacy Questionnaire (K&A) X X
Self-Care and Support Assessment X X
Family Background & Services Survey X X
User Version of the Mobile Health Application Rating Scale (uMARS) X"

6a-i) Online questionnaires: describe if they were validated for online use and apply CHERRIES items to describe how the questionnaires were designed/deployed

If outcomes were obtained through online questionnaires, describe if they were validated for online use and apply CHERRIES items to describe how the questionnaires were designed/deployed [9].

	1	2	3	4	5	
subitem not at all important	○	○	○	○	○	essential

Does your paper address subitem 6a-i?

Copy and paste relevant sections from manuscript text

We pilot tested all surveys in our prior feasibility study (Petrenko et al., 2024) and made revisions to improve performance. Several measures have been previously validated for online use. We provide reliability indices for our study population where relevant.

6a-ii) Describe whether and how “use” (including intensity of use/dosage) was defined/measured/monitored

Describe whether and how “use” (including intensity of use/dosage) was defined/measured/monitored (logins, logfile analysis, etc.). Use/adoption metrics are important process outcomes that should be reported in any ehealth trial.

	1	2	3	4	5	
subitem not at all important	○	○	○	○	○	essential

Does your paper address subitem 6a-ii?

Copy and paste relevant sections from manuscript text

We report usage metrics in table 3 and examine usage patterns by demographics and baseline functioning. We also examine usage and intervention change in the results section.

There is not a defined dosage for the app.

6a-iii) Describe whether, how, and when qualitative feedback from participants was obtained

Describe whether, how, and when qualitative feedback from participants was obtained (e.g., through emails, feedback forms, interviews, focus groups).

	1	2	3	4	5	
subitem not at all important	○	○	○	○	○	essential

Does your paper address subitem 6a-iii?

Copy and paste relevant sections from manuscript text

Qualitative feedback was not obtained in this RCT, but was extensively collected and analyzed in 3 prior studies.
"The systematic user-centered design approach to develop and test the FMF Connect app has been relatively rare in the field of mHealth apps. This approach, detailed in Petrenko et al. (2021), emphasizes iterative feedback at each stage of development from community members who represent target users for the app. This process has included focus groups with caregivers on the initial design and functionalities of the proposed app (Petrenko et al., 2020), interviews with caregivers and providers following beta-testing of initial app prototypes (Petrenko et al., 2021), and surveys and interviews with caregivers during a larger-scale feasibility trial (Petrenko et al., 2024)."
In this study we collected quantitative ratings on the uMARS to assess perceived app quality.

6b) Any changes to trial outcomes after the trial commenced, with reasons

Does your paper address CONSORT subitem 6b? *

Copy and paste relevant sections from the manuscript (include quotes in quotation marks "like this" to indicate direct quotes from your manuscript), or elaborate on this item by providing additional information not in the ms, or briefly explain why the item is not applicable/relevant for your study

"There were no changes in measurement after study initiation."

7a) How sample size was determined

NPT: When applicable, details of whether and how the clustering by care provides or centers was addressed

7a-i) Describe whether and how expected attrition was taken into account when calculating the sample size

Describe whether and how expected attrition was taken into account when calculating the sample size.

	1	2	3	4	5	
subitem not at all important	○	○	○	○	○	essential

Does your paper address subitem 7a-i?

Copy and paste relevant sections from manuscript title (include quotes in quotation marks "like this" to indicate direct quotes from your manuscript), or elaborate on this item by providing additional information not in the ms, or briefly explain why the item is not applicable/relevant for your study

"We aimed to enroll about 150 participants in this study. We anticipated about 20% attribution based on prior feasibility trials."

7b) When applicable, explanation of any interim analyses and stopping guidelines

Does your paper address CONSORT subitem 7b? *

Copy and paste relevant sections from the manuscript (include quotes in quotation marks "like this" to indicate direct quotes from your manuscript), or elaborate on this item by providing additional information not in the ms, or briefly explain why the item is not applicable/relevant for your study

This was not applicable to our study

8a) Method used to generate the random allocation sequence

NPT: When applicable, how care providers were allocated to each trial group

Does your paper address CONSORT subitem 8a? *

Copy and paste relevant sections from the manuscript (include quotes in quotation marks "like this" to indicate direct quotes from your manuscript), or elaborate on this item by providing additional information not in the ms, or briefly explain why the item is not applicable/relevant for your study

"Randomization
Randomization was conducted using REDCap's Randomization package. The allocation sequence was developed by a biostatistician at the University not affiliated with the project to randomly allocate participants equally across the 3 study arms. No stratification or blocking was used. The University Research IT department implemented the allocation table in the REDCap Randomization package and the research team did not have access to the sequence. Once the study coordinator determined a participant was eligible and baseline measures were complete, the study Randomization form would determine the participant's group status. The coordinator then informed the participant of their group status and sent instructions on next steps (i.e., how to install the app, or wait time) via email."

8b) Type of randomisation; details of any restriction (such as blocking and block size)

Does your paper address CONSORT subitem 8b? *

Copy and paste relevant sections from the manuscript (include quotes in quotation marks "like this" to indicate direct quotes from your manuscript), or elaborate on this item by providing additional information not in the ms, or briefly explain why the item is not applicable/relevant for your study

"No stratification or blocking was used."

9) Mechanism used to implement the random allocation sequence (such as sequentially numbered containers), describing any steps taken to conceal the sequence until interventions were assigned

Does your paper address CONSORT subitem 9? *

Copy and paste relevant sections from the manuscript (include quotes in quotation marks "like this" to indicate direct quotes from your manuscript), or elaborate on this item by providing additional information not in the ms, or briefly explain why the item is not applicable/relevant for your study

""Randomization
Randomization was conducted using REDCap's Randomization package. The allocation sequence was developed by a biostatistician at the University not affiliated with the project to randomly allocate participants equally across the 3 study arms. No stratification or blocking was used. The University Research IT department implemented the allocation table in the REDCap Randomization package and the research team did not have access to the sequence. Once the study coordinator determined a participant was eligible and baseline measures were complete, the study Randomization form would determine the participant's group status. The coordinator then informed the participant of their group status and sent instructions on next steps (i.e., how to install the app, or wait time) via email."

10) Who generated the random allocation sequence, who enrolled participants, and who assigned participants to interventions

Does your paper address CONSORT subitem 10? *

Copy and paste relevant sections from the manuscript (include quotes in quotation marks "like this" to indicate direct quotes from your manuscript), or elaborate on this item by providing additional information not in the ms, or briefly explain why the item is not applicable/relevant for your study

"Once the study coordinator determined a participant was eligible and baseline measures were complete, the study Randomization form would determine the participant's group status. The coordinator then informed the participant of their group status and sent instructions on next steps (i.e., how to install the app, or wait time) via email."

11a) If done, who was blinded after assignment to interventions (for example, participants, care providers, those assessing outcomes) and how

NPT: Whether or not administering co-interventions were blinded to group assignment

11a-i) Specify who was blinded, and who wasn't

Specify who was blinded, and who wasn't. Usually, in web-based trials it is not possible to blind the participants [1, 3] (this should be clearly acknowledged), but it may be possible to blind outcome assessors, those doing data analysis or those administering co-interventions (if any).

	1	2	3	4	5	
subitem not at all important	○	○	○	○	○	essential

Does your paper address subitem 11a-i? *

Copy and paste relevant sections from the manuscript (include quotes in quotation marks "like this" to indicate direct quotes from your manuscript), or elaborate on this item by providing additional information not in the ms, or briefly explain why the item is not applicable/relevant for your study

"The research assistant processing survey data and payments could not access the REDCap Randomization package and was blind to group status."

11a-ii) Discuss e.g., whether participants knew which intervention was the "intervention of interest" and which one was the "comparator"

Informed consent procedures (4a-ii) can create biases and certain expectations - discuss e.g., whether participants knew which intervention was the "intervention of interest" and which one was the "comparator".

	1	2	3	4	5	
subitem not at all important	○	○	○	○	○	essential

Does your paper address subitem 11a-ii?

Copy and paste relevant sections from the manuscript (include quotes in quotation marks "like this" to indicate direct quotes from your manuscript), or elaborate on this item by providing additional information not in the ms, or briefly explain why the item is not applicable/relevant for your study

This is not applicable to our study design. The comparator was a waitlist control group. Participants were aware of their group status.

11b) If relevant, description of the similarity of interventions

(this item is usually not relevant for ehealth trials as it refers to similarity of a placebo or sham intervention to a active medication/intervention)

Does your paper address CONSORT subitem 11b? *

Copy and paste relevant sections from the manuscript (include quotes in quotation marks "like this" to indicate direct quotes from your manuscript), or elaborate on this item by providing additional information not in the ms, or briefly explain why the item is not applicable/relevant for your study

This is not applicable for this study

12a) Statistical methods used to compare groups for primary and secondary outcomes

NPT: When applicable, details of whether and how the clustering by care providers or centers was addressed

Does your paper address CONSORT subitem 12a? *

Copy and paste relevant sections from the manuscript (include quotes in quotation marks "like this" to indicate direct quotes from your manuscript), or elaborate on this item by providing additional information not in the ms, or briefly explain why the item is not applicable/relevant for your study

"Intervention change analyses utilized repeated-measures analysis of variance (RMANOVA) for variables collected at the three timepoints. Simultaneous linear regression was utilized for variables collected at baseline and 12-week follow-up. For regressions, group status and baseline levels were entered in Step 1. All regressions were predicting outcomes at 12-week follow-up. False Discovery Rate (FDR) correction was utilized and reported for all regressions. Cohen's d effect sizes were calculated for each outcome to demonstrate the magnitude of the intervention change. Additionally, simultaneous linear regressions were carried out to assess whether app usage within the FMF group predicted 6-week change scores and 12-week change scores."

12a-i) Imputation techniques to deal with attrition / missing values

Imputation techniques to deal with attrition / missing values: Not all participants will use the intervention/comparator as intended and attrition is typically high in ehealth trials. Specify how participants who did not use the application or dropped out from the trial were treated in the statistical analysis (a complete case analysis is strongly discouraged, and simple imputation techniques such as LOCF may also be problematic [4]).

	1	2	3	4	5	
subitem not at all important	○	○	○	○	○	essential

Does your paper address subitem 12a-i? *

Copy and paste relevant sections from the manuscript (include quotes in quotation marks "like this" to indicate direct quotes from your manuscript), or elaborate on this item by providing additional information not in the ms, or briefly explain why the item is not applicable/relevant for your study

"Missing data was primarily handled using average imputation; however, Mplus analysis utilized Full Information Maximum Likelihood (FIML)."

12b) Methods for additional analyses, such as subgroup analyses and adjusted analyses

Does your paper address CONSORT subitem 12b? *

Copy and paste relevant sections from the manuscript (include quotes in quotation marks "like this" to indicate direct quotes from your manuscript), or elaborate on this item by providing additional information not in the ms, or briefly explain why the item is not applicable/relevant for your study

"Point-biserial correlations between group (FMF Connect vs. WLC) and change scores (6-week timepoint - baseline) were conducted to see if mediation was plausible. For those significant correlations, mediation analyses were conducted in Mplus utilizing change scores as the mediator and the ECBI (child behavior) and PSOC (parenting efficacy/satisfaction) as the outcomes, controlling for baseline levels."

X26) REB/IRB Approval and Ethical Considerations [recommended as subheading under "Methods"] (not a CONSORT item)

X26-i) Comment on ethics committee approval

	1	2	3	4	5	
subitem not at all important	○	○	○	○	○	essential

Does your paper address subitem X26-i?

Copy and paste relevant sections from the manuscript (include quotes in quotation marks "like this" to indicate direct quotes from your manuscript), or elaborate on this item by providing additional information not in the ms, or briefly explain why the item is not applicable/relevant for your study

"All study procedures and materials were reviewed and approved by the University Institutional Review Board (STUDY00006555). "

x26-ii) Outline informed consent procedures

Outline informed consent procedures e.g., if consent was obtained offline or online (how? Checkbox, etc.?), and what information was provided (see 4a-ii). See [6] for some items to be included in informed consent documents.

	1	2	3	4	5	
subitem not at all important	○	○	○	○	○	essential

Does your paper address subitem X26-ii?

Copy and paste relevant sections from the manuscript (include quotes in quotation marks "like this" to indicate direct quotes from your manuscript), or elaborate on this item by providing additional information not in the ms, or briefly explain why the item is not applicable/relevant for your study

"All surveys were administered via REDCap, hosted by the University of Rochester, and REDCap's eConsent package was used to document participant's informed consent. Participants had the option to review study consent information by video or text and were required to answer several questions to ensure comprehension. Participants were also offered the opportunity to ask questions via direct contact with the study coordinator before proceeding to other parts of the survey."

X26-iii) Safety and security procedures

Safety and security procedures, incl. privacy considerations, and any steps taken to reduce the likelihood or detection of harm (e.g., education and training, availability of a hotline)

	1	2	3	4	5	
subitem not at all important	○	○	○	○	○	essential

Does your paper address subitem X26-iii?

Copy and paste relevant sections from the manuscript (include quotes in quotation marks "like this" to indicate direct quotes from your manuscript), or elaborate on this item by providing additional information not in the ms, or briefly explain why the item is not applicable/relevant for your study

"Trained peer moderators moderated discussion in family forums to ensure safety."

RESULTS

13a) For each group, the numbers of participants who were randomly assigned, received intended treatment, and were analysed for the primary outcome

NPT: The number of care providers or centers performing the intervention in each group and the number of patients treated by each care provider in each center

Does your paper address CONSORT subitem 13a? *

Copy and paste relevant sections from the manuscript (include quotes in quotation marks "like this" to indicate direct quotes from your manuscript), or elaborate on this item by providing additional information not in the ms, or briefly explain why the item is not applicable/relevant for your study

This is provided in CONSORT diagram, which is Figure 2 in the paper.

13b) For each group, losses and exclusions after randomisation, together with reasons

Does your paper address CONSORT subitem 13b? (NOTE: Preferably, this is shown in a CONSORT flow diagram) *

Copy and paste relevant sections from the manuscript (include quotes in quotation marks "like this" to indicate direct quotes from your manuscript), or elaborate on this item by providing additional information not in the ms, or briefly explain why the item is not applicable/relevant for your study

This is provided in CONSORT diagram, which is Figure 2 in the paper.

13b-i) Attrition diagram

Strongly recommended: An attrition diagram (e.g., proportion of participants still logging in or using the intervention/comparator in each group plotted over time, similar to a survival curve) or other figures or tables demonstrating usage/dose/engagement.

	1	2	3	4	5	
subitem not at all important	○	○	○	○	○	essential

Does your paper address subitem 13b-i?

Copy and paste relevant sections from the manuscript or cite the figure number if applicable (include quotes in quotation marks "like this" to indicate direct quotes from your manuscript), or elaborate on this item by providing additional information not in the ms, or briefly explain why the item is not applicable/relevant for your study

Table 3 summarizes app use indicators for this study.
An attrition diagram was not included in this paper, but was examined in a prior feasibility trial of the app.

14a) Dates defining the periods of recruitment and follow-up

Does your paper address CONSORT subitem 14a? *

Copy and paste relevant sections from the manuscript (include quotes in quotation marks "like this" to indicate direct quotes from your manuscript), or elaborate on this item by providing additional information not in the ms, or briefly explain why the item is not applicable/relevant for your study

"Recruitment for the current study began on January 6, 2022 with enrollment ending August 31, 2022. The study was completed on December 31, 2022 after all participants had the opportunity to complete follow-up surveys."

14a-i) Indicate if critical "secular events" fell into the study period

Indicate if critical "secular events" fell into the study period, e.g., significant changes in Internet resources available or "changes in computer hardware or Internet delivery resources"

	1	2	3	4	5	
subitem not at all important	○	○	○	○	○	essential

Does your paper address subitem 14a-i?

Copy and paste relevant sections from the manuscript (include quotes in quotation marks "like this" to indicate direct quotes from your manuscript), or elaborate on this item by providing additional information not in the ms, or briefly explain why the item is not applicable/relevant for your study

This was not applicable for this study

14b) Why the trial ended or was stopped (early)

Does your paper address CONSORT subitem 14b? *

Copy and paste relevant sections from the manuscript (include quotes in quotation marks "like this" to indicate direct quotes from your manuscript), or elaborate on this item by providing additional information not in the ms, or briefly explain why the item is not applicable/relevant for your study

This was not applicable for this study

15) A table showing baseline demographic and clinical characteristics for each group

NPT: When applicable, a description of care providers (case volume, qualification, expertise, etc.) and centers (volume) in each group

Does your paper address CONSORT subitem 15? *

Copy and paste relevant sections from the manuscript (include quotes in quotation marks "like this" to indicate direct quotes from your manuscript), or elaborate on this item by providing additional information not in the ms, or briefly explain why the item is not applicable/relevant for your study

This is provided in Table 2

15-i) Report demographics associated with digital divide issues

In ehealth trials it is particularly important to report demographics associated with digital divide issues, such as age, education, gender, social-economic status, computer/Internet/ehealth literacy of the participants, if known.

	1	2	3	4	5	
subitem not at all important	○	○	○	○	○	essential

Does your paper address subitem 15-i? *

Copy and paste relevant sections from the manuscript (include quotes in quotation marks "like this" to indicate direct quotes from your manuscript), or elaborate on this item by providing additional information not in the ms, or briefly explain why the item is not applicable/relevant for your study

Table 2 reports participant demographics. These are examined relative to study attrition, and app usage in the study.
"One-way ANOVAs (continuous variables) and Pearson Chi-Square (categorical variables) analyses were utilized to determine if group differences by demographic factors were present. There were no significant differences between the FMF Connect + Coaching, FMF Connect, and WLC groups on any continuous variables (age, income, household structure), nor were there significant group differences among the majority of categorical variables (gender, race, ethnicity, relationship to child). There was a significant group difference on variables characterizing caregiver marital status. However, given the small size of marital status subgroups, this variable was not controlled for in further analyses. Given the absence of group differences, no other demographic factors were controlled for. "
"The trial CONSORT diagram is provided (Figure 2). There were no significant differences in demographic characteristics (caregiver gender, caregiver race/ethnicity, caregiver education level, caregiver education level, comfort with technology, knowledge of FASD, income, child gender, child race/ethnicity, caregiver type) between those who did and did not complete data collection at the 6-week or 12-week timepoints. Caregiver age and child age were not correlated with attrition. T-tests resulted in no significant difference in attrition by number of children in the home."

16) For each group, number of participants (denominator) included in each analysis and whether the analysis was by original assigned groups

16-i) Report multiple “denominators” and provide definitions

Report multiple “denominators” and provide definitions: Report N’s (and effect sizes) “across a range of study participation [and use] thresholds” [1], e.g., N exposed, N consented, N used more than x times, N used more than y weeks, N participants “used” the intervention/comparator at specific pre-defined time points of interest (in absolute and relative numbers per group). Always clearly define “use” of the intervention.

	1	2	3	4	5	
subitem not at all important	○	○	○	○	○	essential

Does your paper address subitem 16-i? *

Copy and paste relevant sections from the manuscript (include quotes in quotation marks "like this" to indicate direct quotes from your manuscript), or elaborate on this item by providing additional information not in the ms, or briefly explain why the item is not applicable/relevant for your study

"All analyses use the full intent-to-treat sample and involve two-sided hypothesis testing." Data tables include number of participants with data and/or degrees of freedom in analyses.

16-ii) Primary analysis should be intent-to-treat

Primary analysis should be intent-to-treat, secondary analyses could include comparing only "users", with the appropriate caveats that this is no longer a randomized sample (see 18-i).

	1	2	3	4	5	
subitem not at all important	○	○	○	○	○	essential

Does your paper address subitem 16-ii?

Copy and paste relevant sections from the manuscript (include quotes in quotation marks "like this" to indicate direct quotes from your manuscript), or elaborate on this item by providing additional information not in the ms, or briefly explain why the item is not applicable/relevant for your study

"All analyses use the full intent-to-treat sample and involve two-sided hypothesis testing."

17a) For each primary and secondary outcome, results for each group, and the estimated effect size and its precision (such as 95% confidence interval)

Does your paper address CONSORT subitem 17a? *

Copy and paste relevant sections from the manuscript (include quotes in quotation marks "like this" to indicate direct quotes from your manuscript), or elaborate on this item by providing additional information not in the ms, or briefly explain why the item is not applicable/relevant for your study

Cohen's D and 95% CIs are provided in Table 6

17a-i) Presentation of process outcomes such as metrics of use and intensity of use

In addition to primary/secondary (clinical) outcomes, the presentation of process outcomes such as metrics of use and intensity of use (dose, exposure) and their operational definitions is critical. This does not only refer to metrics of attrition (13-b) (often a binary variable), but also to more continuous exposure metrics such as “average session length”. These must be accompanied by a technical description how a metric like a “session” is defined (e.g., timeout after idle time) [1] (report under item 6a).

	1	2	3	4	5	
subitem not at all important	○	○	○	○	○	essential

Does your paper address subitem 17a-i?

Copy and paste relevant sections from the manuscript (include quotes in quotation marks "like this" to indicate direct quotes from your manuscript), or elaborate on this item by providing additional information not in the ms, or briefly explain why the item is not applicable/relevant for your study

App usage and intervention change were examined.
"Learning module completion and app opening were entered into a simultaneous regression model to predict change scores at the 6-week and 12-week timepoints (Multimedia Appendix 2). Learning module completion had an effect on PSOC efficacy change scores over 12 weeks (b=-.307, p=.029), in that those who completed more learning modules had greater levels of parenting efficacy after 12 weeks. Caregivers who opened the app more showed more improvement on RCB emotion seeking at the 12-week timepoint (b=.307, p=.030) and PSOC Efficacy at the 6-week timepoint (b=.214, p=.004)."

17b) For binary outcomes, presentation of both absolute and relative effect sizes is recommended

Does your paper address CONSORT subitem 17b? *

Copy and paste relevant sections from the manuscript (include quotes in quotation marks "like this" to indicate direct quotes from your manuscript), or elaborate on this item by providing additional information not in the ms, or briefly explain why the item is not applicable/relevant for your study

We did not have any binary primary outcomes.

18) Results of any other analyses performed, including subgroup analyses and adjusted analyses, distinguishing pre-specified from exploratory

Does your paper address CONSORT subitem 18? *

Copy and paste relevant sections from the manuscript (include quotes in quotation marks "like this" to indicate direct quotes from your manuscript), or elaborate on this item by providing additional information not in the ms, or briefly explain why the item is not applicable/relevant for your study

Pairwise comparisons were used to follow up significant interactions.
False Discovery Rate values were provided given multiple comparisons.

18-i) Subgroup analysis of comparing only users

A subgroup analysis of comparing only users is not uncommon in ehealth trials, but if done, it must be stressed that this is a self-selected sample and no longer an unbiased sample from a randomized trial (see 16-iii).

	1	2	3	4	5	
subitem not at all important	○	○	○	○	○	essential

Does your paper address subitem 18-i?

Copy and paste relevant sections from the manuscript (include quotes in quotation marks "like this" to indicate direct quotes from your manuscript), or elaborate on this item by providing additional information not in the ms, or briefly explain why the item is not applicable/relevant for your study

This was not used in this study. All analyses were intent to treat

19) All important harms or unintended effects in each group
(for specific guidance see CONSORT for harms)

Does your paper address CONSORT subitem 19? *

Copy and paste relevant sections from the manuscript (include quotes in quotation marks "like this" to indicate direct quotes from your manuscript), or elaborate on this item by providing additional information not in the ms, or briefly explain why the item is not applicable/relevant for your study

"No harms or unintended effects were identified for participants."

19-i) Include privacy breaches, technical problems

Include privacy breaches, technical problems. This does not only include physical “harm” to participants, but also incidents such as perceived or real privacy breaches [1], technical problems, and other unexpected/unintended incidents. “Unintended effects” also includes unintended positive effects [2].

	1	2	3	4	5	
subitem not at all important	○	○	○	○	○	essential

Does your paper address subitem 19-i?

Copy and paste relevant sections from the manuscript (include quotes in quotation marks "like this" to indicate direct quotes from your manuscript), or elaborate on this item by providing additional information not in the ms, or briefly explain why the item is not applicable/relevant for your study

This was not applicable for this study.

19-ii) Include qualitative feedback from participants or observations from staff/researchers

Include qualitative feedback from participants or observations from staff/researchers, if available, on strengths and shortcomings of the application, especially if they point to unintended/unexpected effects or uses. This includes (if available) reasons for why people did or did not use the application as intended by the developers.

	1	2	3	4	5	
subitem not at all important	○	○	○	○	○	essential

Does your paper address subitem 19-ii?

Copy and paste relevant sections from the manuscript (include quotes in quotation marks "like this" to indicate direct quotes from your manuscript), or elaborate on this item by providing additional information not in the ms, or briefly explain why the item is not applicable/relevant for your study

This was an extensive focus of prior development studies. The current study did not include qualitative feedback.

DISCUSSION

22) Interpretation consistent with results, balancing benefits and harms, and considering other relevant evidence

NPT: In addition, take into account the choice of the comparator, lack of or partial blinding, and unequal expertise of care providers or centers in each group

22-i) Restate study questions and summarize the answers suggested by the data, starting with primary outcomes and process outcomes (use)

Restate study questions and summarize the answers suggested by the data, starting with primary outcomes and process outcomes (use).

	1	2	3	4	5	
subitem not at all important	○	○	○	○	○	essential

Does your paper address subitem 22-i? *

Copy and paste relevant sections from the manuscript (include quotes in quotation marks "like this" to indicate direct quotes from your manuscript), or elaborate on this item by providing additional information not in the ms, or briefly explain why the item is not applicable/relevant for your study

"Families raising children with FASD experience poor access to evidence-based care in their communities given multiple systems-level barriers. These include limited professional training in FASD, long waitlists and provider shortages, and pervasive stigma. Complementing efforts to build workforce capacity, the current study tested the efficacy of FMF Connect, a first of its kind: a caregiver self-directed mHealth intervention. Results from this RCT provide initial support for the efficacy of the FMF Connect app for caregivers raising preschool and school-aged children with FASD or PAE. Relative to the waitlist group, groups that received the FMF Connect app had greater reductions in willful attributions of child behavior, increased parenting satisfaction, and more family needs met. These changes in caregiver outcomes are notable given the low intensity, self-directed nature of the intervention. Contrary to hypotheses, no significant group differences were found for child outcomes assessed. Consistent with our prior feasibility trial (Petrenko et al., 2024), caregivers gave high ratings for app quality, with the quality of information rated highest among indicators. App usage was relatively greater for caregivers reporting more difficulties with parenting efficacy, completing daily tasks with their children, and meeting family needs."

22-ii) Highlight unanswered new questions, suggest future research

Highlight unanswered new questions, suggest future research.

	1	2	3	4	5	
subitem not at all important	○	○	○	○	○	essential

Does your paper address subitem 22-ii?

Copy and paste relevant sections from the manuscript (include quotes in quotation marks "like this" to indicate direct quotes from your manuscript), or elaborate on this item by providing additional information not in the ms, or briefly explain why the item is not applicable/relevant for your study

"Future larger-scale investigations may allow for greater power and ability to utilize machine learning techniques to explore usage patterns in more nuanced ways. Finally, based on the theorized logic model for the FMF Program, this study hypothesized that caregiver attributions of behavior would mediate changes in caregiver efficacy and satisfaction and child behavior outcomes. Results of this study did not support this hypothesis. While intervention group was correlated with 6-week change on two scales, mediation was not significant. In addition, while intervention effects were seen for parenting satisfaction, the app was not associated with change in parenting efficacy or child behavior over the 12-week evaluation period. Given intervention effects are seen on these outcomes in the longer duration follow-ups for the specialist-led FMF Program (same FMF cites as above), it is possible more intensive intervention and/or follow-up is needed for these outcomes."

20) Trial limitations, addressing sources of potential bias, imprecision, and, if relevant, multiplicity of analyses

20-i) Typical limitations in ehealth trials

Typical limitations in ehealth trials: Participants in ehealth trials are rarely blinded. Ehealth trials often look at a multiplicity of outcomes, increasing risk for a Type I error. Discuss biases due to non-use of the intervention/usability issues, biases through informed consent procedures, unexpected events.

	1	2	3	4	5	
subitem not at all important	○	○	○	○	○	essential

Does your paper address subitem 20-i? *

Copy and paste relevant sections from the manuscript (include quotes in quotation marks "like this" to indicate direct quotes from your manuscript), or elaborate on this item by providing additional information not in the ms, or briefly explain why the item is not applicable/relevant for your study

"enrolled participants were largely white adoptive mothers. Caregiver education and income were also higher than national averages. Our study was also limited to participants with Apple iOS devices, which may have influenced patterns of participant demographics."
"As with most behavioral intervention studies, participants were aware of their treatment condition, which could have potentially biased follow-up responses. FMF Connect targeted multiple child and family outcomes. As a result, multiple analyses were run to assess the range of possible intervention effects targeted. The number of analyses may have increased the likelihood of false positive intervention effects, as suggested by FDR corrections."

21) Generalisability (external validity, applicability) of the trial findings

NPT: External validity of the trial findings according to the intervention, comparators, patients, and care providers or centers involved in the trial

21-i) Generalizability to other populations

Generalizability to other populations: In particular, discuss generalizability to a general Internet population, outside of a RCT setting, and general patient population, including applicability of the study results for other organizations

	1	2	3	4	5	
subitem not at all important	○	○	○	○	○	essential

Does your paper address subitem 21-i?

Copy and paste relevant sections from the manuscript (include quotes in quotation marks "like this" to indicate direct quotes from your manuscript), or elaborate on this item by providing additional information not in the ms, or briefly explain why the item is not applicable/relevant for your study

"The generalizability of study findings may well be impacted by characteristics of study participants who enrolled in the study and other design features. ..."

21-ii) Discuss if there were elements in the RCT that would be different in a routine application setting

Discuss if there were elements in the RCT that would be different in a routine application setting (e.g., prompts/reminders, more human involvement, training sessions or other co-interventions) and what impact the omission of these elements could have on use, adoption, or outcomes if the intervention is applied outside of a RCT setting.

	1	2	3	4	5	
subitem not at all important	○	○	○	○	○	essential

Does your paper address subitem 21-ii?

Copy and paste relevant sections from the manuscript (include quotes in quotation marks "like this" to indicate direct quotes from your manuscript), or elaborate on this item by providing additional information not in the ms, or briefly explain why the item is not applicable/relevant for your study

This is not applicable to our study.

OTHER INFORMATION

23) Registration number and name of trial registry

Does your paper address CONSORT subitem 23? *

Copy and paste relevant sections from the manuscript (include quotes in quotation marks "like this" to indicate direct quotes from your manuscript), or elaborate on this item by providing additional information not in the ms, or briefly explain why the item is not applicable/relevant for your study

ClinicalTrials.gov (NCT05028517)

24) Where the full trial protocol can be accessed, if available

Does your paper address CONSORT subitem 24? *

Cite a Multimedia Appendix, other reference, or copy and paste relevant sections from the manuscript (include quotes in quotation marks "like this" to indicate direct quotes from your manuscript), or elaborate on this item by providing additional information not in the ms, or briefly explain why the item is not applicable/relevant for your study

ClinicalTrials.gov (NCT05028517)

25) Sources of funding and other support (such as supply of drugs), role of funders

Does your paper address CONSORT subitem 25? *

Copy and paste relevant sections from the manuscript (include quotes in quotation marks "like this" to indicate direct quotes from your manuscript), or elaborate on this item by providing additional information not in the ms, or briefly explain why the item is not applicable/relevant for your study

"All or part of this work was done in conjunction with the Collaborative Initiative on Fetal Alcohol Spectrum Disorders (CIFASD, https://doi.org/10.5967/ntw9-h991), which is funded by grants from the National Institute on Alcohol Abuse and Alcoholism (NIAAA). Additional information about CIFASD, including information describing available data, can be found at www.cifasd.org. The data presented in this manuscript was specifically funded under grant U01AA026104 "Development and Evaluation of an Evidence-Based Mobile Health Caregiver Intervention for FASD." Data from this study are part of CIFASD Phase 4 (2017-2022)."

X27) Conflicts of Interest (not a CONSORT item)

X27-i) State the relation of the study team towards the system being evaluated

In addition to the usual declaration of interests (financial or otherwise), also state the relation of the study team towards the system being evaluated, i.e., state if the authors/evaluators are distinct from or identical with the developers/sponsors of the intervention.

	1	2	3	4	5	
subitem not at all important	○	○	○	○	○	essential

Does your paper address subitem X27-i?

Copy and paste relevant sections from the manuscript (include quotes in quotation marks "like this" to indicate direct quotes from your manuscript), or elaborate on this item by providing additional information not in the ms, or briefly explain why the item is not applicable/relevant for your study

Conflicts of Interest
None declared.

About the CONSORT EHEALTH checklist

As a result of using this checklist, did you make changes in your manuscript? *

- ○ yes, major changes
- ◉ yes, minor changes
- ○ no

What were the most important changes you made as a result of using this checklist?

Your answer

How much time did you spend on going through the checklist INCLUDING making changes in your manuscript *

It took about 1.5 - 2 hours to do in one sitting.

As a result of using this checklist, do you think your manuscript has improved? *

- (x) yes
- ( ) no
- ( ) Other:

Would you like to become involved in the CONSORT EHEALTH group?

This would involve for example becoming involved in participating in a workshop and writing an "Explanation and Elaboration" document

- ( ) yes
- (x) no
- ( ) Other:

Clear selection

Any other comments or questions on CONSORT EHEALTH

It would be helpful to have this in a format that was easy to save and come back to and work on in stages. It was stressful to have to do in a single setting.

STOP - Save this form as PDF before you click submit

To generate a record that you filled in this form, we recommend to generate a PDF of this page (on a Mac, simply select "print" and then select "print as PDF") before you submit it.

When you submit your (revised) paper to JMIR, please upload the PDF as supplementary file.

Don't worry if some text in the textboxes is cut off, as we still have the complete information in our database. Thank you!

Final step: Click submit !

Click submit so we have your answers in our database!

Submit

Clear form

Never submit passwords through Google Forms.

This content is neither created nor endorsed by Google. - Terms of Service - Privacy Policy

Does this form look suspicious? Report

Google Forms
